# Supplementary material for: Acceptance of COVID-19 vaccines in sub-Saharan Africa: evidence from six national phone surveys
Source: BMJ Open. 2021 Dec 15;11(12):e055159. doi: 10.1136/bmjopen-2021-055159 (PMC8678558; doi:10.1136/bmjopen-2021-055159)
Supplement: Supplementary data [file bmjopen-2021-055159supp001.pdf]

Appendix

1. Tables

Table A.1. Household selection, number of households

Panel A. Households (N)	**Burkina Faso**	**Ethiopia**	**Malawi**	**Mali**	**Nigeria**	**Uganda**
Pre-COVID-19 FtF survey	7,010	6,770	3,181	7,000	4,976	3,098
With phone numbers	6,877	5,374	2,337	6,300	4,934	2,386
Attempted to contact	2,500	5,374	2,337	2,270	3,000	2,386
Reached	2,062	3,357	1,743	2,270	2,057	2,246
Phone interviews completed	1,945	2,701	1,589	1,765	1,766	2,129

Table A. 2. Unweighted descriptive statistics in the baseline F2F surveys and the HFPS .

	**Burkina Faso**		**Ethiopia**		**Malawi**		**Mali**		**Nigeria**		**Uganda**	
	F2F	HFPS	F2F	HFPS	F2F	HFPS	F2F	HFPS	F2F	HFPS	F2F	HFPS
Men	45.5	78.7	47.3	62.7	47.6	59.8	47.4	78.9	48.3	72.3	48.2	50.7
HH heads	28.7	86.1	38.5	82.5	37.0	74.5	27.1	82.3	32.7	79.3	35.1	74.5
Urban	44.9	72.0	54.0	71.9	26.0	36.8	41.7	66.3	32.0	39.3	24.2	25.8
Primary education	26.1	32.4	34.6	52.9	37.2	50.2	20.2	30.9	66.5	75.1	47.8	51.1
< 30 years	44.1	11.3	49.6	29.4	52.9	27.8	42.6	13.8	42.5	13.5	48.2	15.3
30 - 60 years	45.7	72.2	42.1	62.4	38.2	62.7	45.7	70.4	44.9	69.0	40.0	66.2
60+ years	10.1	16.5	8.3	8.3	8.9	9.5	11.7	15.9	12.6	17.5	11.8	18.6
Quintile 1 (poorest)	18.7	14.0	11.4	4.6	15.7	8.7	18.2	14.6	12.7	9.5	18.2	15.8
Quintile 2	18.9	15.6	13.8	9.5	17.6	14.6	18.9	16.4	14.4	13.1	19.0	18.5
Quintile 3	19.9	17.9	15.0	12.4	17.4	16.1	21.0	19.0	19.2	18.7	20.4	20.7
Quintile 4	21.3	22.5	20.3	22.0	22.2	23.5	20.7	22.2	21.4	21.9	21.7	22.5
Quintile 5 (richest)	21.2	30.0	39.4	51.5	27.1	37.1	21.2	27.8	32.3	36.9	20.7	22.5
**Observations**	24,396	1,742	17,563	2,701	8,588	1,589	24,394	1,765	15,230	1,766	8,763	2,129

Note: The table shows descriptive statistics and sample sizes for the face-to-face (F2F) pre-COVID samples of the general adult population (>15 years) and the high-frequency phone surveys (HFPS) during the pandemic. All values except observation numbers in percent.

Table A.3. Statistical significance of cross-country differences in acceptance rates in pairwise, bivariate regressions.

**BFA**						
**ETH**	0.000***					
**MWI**	0.089*	0.000***				
**MLI**	0.000***	0.000***	0.000***			
**NIG**	0.000***	0.000***	0.056*	0.000***		
**UGA**	0.005***	0.000***	0.324	0.000***	0.309	
	**BFA**	**ETH**	**MWI**	**MLI**	**NIG**	**UGA**

Note: P-values from bivariate OLS regressions of vaccine acceptance on a single country dummy using samples consisting of each respective country pair. ***p<0.01, **p<0.05, *p<0.1

Table A.4. Willingness to get vaccinated by country and pooled, mean and 95% confidence interval

If an approved vaccine to prevent coronavirus was available right now at no cost, would you agree to be vaccinated? —Yes	Mean	Lower Bound	Upper Bound	N
Burkina Faso, weighted	79.6%	77.0%	82.3%	1,945
Ethiopia, weighted	97.9%	97.2%	98.6%	2,701
Malawi, weighted	82.7%	80.0%	85.4%	1,589
Mali, weighted	64.5%	61.3%	67.8%	1,765
Nigeria, weighted	86.2%	83.9%	88.5%	1,766
Uganda, weighted	84.5%	82.2%	86.8%	2,129
Pooled, weighted	87.6%	86.4%	88.8%	11,895
Pooled, unweighted	82.4%	81.7%	83.1%	

Table A.5: Bivariate logistic regression on government trust and satisfaction using household-level survey weights.

VARIABLES	(1)<br>Malawi	(2)<br>Mali	(3)<br>Nigeria
Government not trustworthy	-<br>0.0638**		
	(0.0322)		
Satisfied with government response		0.161***	0.0568**
		(0.0552)	(0.0232)
Observations	1,589	1,631	1,766
Controls	YES	YES	YES
Pseudo R2	0.00574	0.00742	0.00847

Robust standard errors in parentheses. *** p<0.01, ** p<0.05, * p<0.1

Kanyanda S, *et al. BMJ Open* 2021; 11:e055159. doi: 10.1136/bmjopen-2021-055159

Table A.6. Estimated acceptance rates using household or individual weights.

	**Burkina Faso**		**Ethiopia**		**Malawi**		**Mali**		**Nigeria**		**Uganda**	
	HH	Indiv.	HH	Indiv.	HH	Indiv.	HH	Indiv.	HH	Indiv.	HH	Indiv.
Overall	0.798	0.815	0.979	0.973	0.827	0.828	0.645	0.657	0.862	0.843	0.845	0.844
	(0.0135)	(0.0176)	(0.0036)	(0.0068)	(0.0137)	(0.0176)	(0.0166)	(0.0241)	(0.0118)	(0.0168)	(0.0116)	(0.0129)
Men	0.800	0.839	0.987	0.987	0.846	0.843	0.642	0.666	0.901	0.888	0.854	0.862
	(0.0151)	(0.0178)	(0.0037)	(0.0050)	(0.0162)	(0.0216)	(0.0185)	(0.0281)	(0.0126)	(0.0200)	(0.0160)	(0.0161)
Women	0.788	0.779	0.958	0.949	0.798	0.816	0.657	0.643	0.757	0.793	0.835	0.831
	(0.0304)	(0.0346)	(0.0088)	(0.0164)	(0.0240)	(0.0268)	(0.0374)	(0.0435)	(0.0269)	(0.0282)	(0.0169)	(0.0191)
Urban	0.653	0.695	0.954	0.947	0.700	0.719	0.669	0.668	0.820	0.814	0.810	0.802
	(0.0207)	(0.0298)	(0.0070)	(0.0131)	(0.0318)	(0.0398)	(0.0156)	(0.0324)	(0.0229)	(0.0302)	(0.0232)	(0.0260)
Rural	0.891	0.905	0.991	0.986	0.859	0.859	0.635	0.650	0.881	0.858	0.861	0.862
	(0.0160)	(0.0181)	(0.0040)	(0.0078)	(0.0148)	(0.0193)	(0.0227)	(0.0335)	(0.0135)	(0.0203)	(0.0132)	(0.0147)
Q1	0.914	0.928	0.997	0.997	0.903	0.922	0.640	0.643	0.949	0.957	0.941	0.956
	(0.0237)	(0.0226)	(0.0023)	(0.0020)	(0.0335)	(0.0280)	(0.0382)	(0.0572)	(0.0244)	(0.0250)	(0.0163)	(0.0120)
Q2	0.914	0.923	0.997	0.994	0.923	0.906	0.703	0.690	0.884	0.849	0.905	0.863
	(0.0224)	(0.0309)	(0.0029)	(0.0055)	(0.0183)	(0.0284)	(0.0347)	(0.0582)	(0.0340)	(0.0493)	(0.0202)	(0.0321)
Q3	0.823	0.812	0.971	0.958	0.819	0.775	0.702	0.699	0.911	0.851	0.849	0.856
	(0.0312)	(0.0406)	(0.0136)	(0.0249)	(0.0329)	(0.0549)	(0.0348)	(0.0552)	(0.0196)	(0.0416)	(0.0263)	(0.0263)
Q4	0.800	0.766	0.986	0.984	0.745	0.779	0.602	0.664	0.834	0.840	0.785	0.784
	(0.0304)	(0.0466)	(0.0048)	(0.0052)	(0.0356)	(0.0400)	(0.0392)	(0.0478)	(0.0286)	(0.0334)	(0.0294)	(0.0324)
Q5	0.548	0.606	0.957	0.949	0.797	0.819	0.582	0.594	0.809	0.798	0.799	0.796
	(0.0312)	(0.0477)	(0.0082)	(0.0171)	(0.0256)	(0.0282)	(0.0361)	(0.0484)	(0.0218)	(0.0304)	(0.0249)	(0.0282)

Note: The table compares estimated acceptance rates for a free, approved COVID-19 vaccine for different demographics. It distinguishes between using weights that correct for coverage and non-response bias at the household-level (household weights) and, where available, using individual-level phone weights that also correct for respondent selection at the individual-level (individual weights). The samples do not comprise observations for which no individual weight could be calibrated due to missing baseline information. Standard errors in parentheses.

Table A.7. Logistic regression on the correlates of hesitancy using individual-level survey weights, marginal effects.

VARIABLES	(1)<br>Burkina Faso	(2)<br>Ethiopia	(3)<br>Malawi	(4)<br>Mali	(5)<br>Nigeria	(6)<br>Uganda
Respondent is male	0.0265	0.0125	0.0272	0.116**	0.0307	0.00464
	(0.0351)	(0.00942)	(0.0371)	(0.0494)	(0.0406)	(0.0244)
Lives in urban area	-0.0750**	-0.0236**	-0.0930***	-0.0221	-0.00481	0.00853
	(0.0323)	(0.0116)	(0.0348)	(0.0388)	(0.0370)	(0.0223)
Years of education	-0.00462	-0.00154	-0.0123***	-0.00337	-0.00657	-0.00261
	(0.00293)	(0.000938)	(0.00417)	(0.00356)	(0.00410)	(0.00247)
Age group, 30-60 yrs	0.0354	-0.00114	-0.00707	-0.0117	-0.0124	-0.0340
	(0.0490)	(0.00984)	(0.0387)	(0.0476)	(0.0356)	(0.0294)
Age group, 60+ yrs	0.124**	0.00134	-0.0331	-0.0471	-0.0321	0.0134
	(0.0491)	(0.0196)	(0.0720)	(0.0612)	(0.0628)	(0.0317)
Household head	-0.00843	0.0226*	-0.00400	-0.0972*	0.0333	0.0204
	(0.0401)	(0.0119)	(0.0385)	(0.0549)	(0.0415)	(0.0267)
Household Size	0.00287	0.00186	0.00971	-0.00534*	0.00834	0.00268
	(0.00510)	(0.00181)	(0.00842)	(0.00322)	(0.00591)	(0.00564)
Expenditure, 2nd quintile	0.00107	0.00591	-0.0203	0.0270	-0.0994	-0.0871***
	(0.0502)	(0.0131)	(0.0469)	(0.0638)	(0.0757)	(0.0323)
Expenditure, 3rd quintile	-0.0660	-0.0277	-0.178**	0.0325	-0.0371	-0.0813***
	(0.0428)	(0.0193)	(0.0712)	(0.0537)	(0.0666)	(0.0287)
Expenditure, 4th quintile	-0.0921**	-0.00480	-0.118**	-0.0504	-0.0428	-0.122***
	(0.0466)	(0.0118)	(0.0522)	(0.0595)	(0.0588)	(0.0316)
Expenditure, 5th (richest) quintile	-0.123**	-0.0157	-0.0143	-0.115*	-0.0679	-0.103***
	(0.0528)	(0.0126)	(0.0461)	(0.0684)	(0.0631)	(0.0317)
Willing to be tested for COVID-19	0.271***	0.0839***	0.249***	0.468***	0.224***	0.348***
	(0.0287)	(0.0149)	(0.0440)	(0.0171)	(0.0302)	(0.0287)
HH received assistance during COVID-19	0.0840*	0.0177	-0.0412	0.0406	-0.00112	0.0597***
	(0.0505)	(0.0127)	(0.0455)	(0.0598)	(0.0328)	(0.0214)
Gov't not trustworthy			-0.0408			
			(0.0322)			
Satisfied with gov't response				0.0356	0.0235	
				(0.0446)	(0.0325)	
Observations	1,738	2,654	1,542	1,590	1,700	2,106
Pseudo R2	0.225	0.357	0.136	0.325	0.162	0.238

Robust standard errors in parentheses. *** p<0.01, ** p<0.05, * p<0.1. Weighted logit regressions with willingness to take a free, approved COVID-19 vaccine as dependent variable.

2. Conception and implementation of the High-Frequency Phone Surveys (HFPS)

The High Frequency Phone Surveys (HFPS) from which we take our data were conceived in March 2020 by a working group formed within the World Bank. They are multi-topic, longitudinal surveys consisting of (i) core modules with core questions that are repeated in each survey round, (ii) optional modules for which inclusion was within the discretion of NSOs according to country need and context, (iii) rotating modules with questions that are only periodically featured in specific survey rounds. The questions on vaccine acceptance belong to the latter category of rotating questions and at the time of writing had only been featured once in the fall of 2020.

In order to safeguard cross-country comparability and maintain the same high standard across implementing NSOs, dedicated working groups within the World Bank elaborated standardized guidelines for sampling [1], questionnaire design (guidelines [2], questionnaire template [3], and interviewer manual [4]), and implementation [5]. These guidelines were circulated ahead of the commencement of the surveys and peer-reviewed inside and outside the World Bank.

The countries covered in our study represent all countries that had their HFPS supported by the Living Standards Measurement Study team within the World Bank.[6] These six countries were the ones where rich, pre-COVID, and cross-country comparable data from the LSMS-ISA household surveys that served as sampling frames existed. With regard to the LSMS-supported HFPS, Carletto and Kilic (2020) provide a detailed discussion of their conception and features.[7]

To select respondents for the HFPS, the pre-COVID LSMS-ISA household surveys [8] served as sampling frames. The pre-COVID LSMS-ISA surveys attempted to collect phone numbers from all household members (or alternatively a reference contact such as a neighbor). The sampling for the HFPS started with all enumeration areas covered in the latest pre-COVID LSMS-ISA household survey. Depending on budget availability, either all households with at least one phone number for a household member or reference contact (Ethiopia, Malawi, and Uganda) or a random sub-sample (Burkina Faso, Nigeria, and Mali) was selected for interviewing. Chapter 2.1 of Brubaker et al. (2021) contains a country-by-country discussion of contact protocols.[9] The target sample size for the HFPS was between 1,500 and 1,800 households, a figure that the survey rounds we take our data on vaccine willingness from all meet or exceed (see Table A.1). The target sample size was calculated in order to ensure that, at a minimum, it would be sufficient to detect a 10 percentage point change in the key indicators (COVID-19 knowledge and behavior and labor market impacts) in between rounds with 90% power and 95% confidence at the national level. Notably, sample size requirements for reliable detection of changes across survey rounds are substantially *higher* than what is required for reliable point estimates within a single round as in the case of our study. A more detailed discussion of the sampling process is provided in Himelein et al. (2020).[1]

To ensure smooth implementation and common standards across countries, the launch of the first round of HFPS was proceeded by three days of piloting the questionnaire, CATI technology, survey protocols, and monitoring mechanisms (for more information, see [5]). Questionnaire guidelines and templates were first elaborated by the World Bank's COVID-19 questionnaire working group and served as the backbone of the HFPS implemented in each NSO (see [2–4]). The country-specific questionnaires implemented in the respective survey round from which our

data on vaccine acceptance stem are openly available together with the data in the World Bank's Micro Data Library.

Interviewers for the survey were selected out of a pool of existing enumerators with experience conducting the LSMS-ISA household surveys. As such, all enumerators had undergone previous LSMS training and were intimately familiar with LSMS-style surveys. In some of the countries, enumerators furthermore had previous experience conducting surveys over the phone. To prepare enumerators specifically for conducting the HFPS, all interviewers received three days of standardized training ahead of the first round of the survey. Additionally, interviewers received a one-day follow-up training in between survey rounds. Regular audio audits ensured consistently high quality between interviewers. The guideline interview manual shared with enumerators across countries can be accessed at [4].

References

1 Himelein K, Eckman S, Kastelic J, *et al.* High Frequency Mobile Phone Surveys of Households to Assess the Impacts of COVID-19. Guidelines on Sampling Design. Washington D.C.: : World Bank 2020. http://documents.worldbank.org/curated/en/742581588695955271/Guidelines-on-Sampling-Design

2 Palacios-Lopez A, Temgoua Noumedem C, Kokas D, *et al.* High Frequency Mobile Phone Surveys of Households to Assess the Impacts of COVID-19 (Vol. 5) : Overview of the Questionnaire Template (English). Washington, D.C.: : World Bank 2020. https://documents.worldbank.org/en/publication/documents-reports/documentdetail/908001588697965879/overview-of-the-questionnaire-template

3 World Bank. High Frequency Mobile Phone Surveys of Households to Assess the Impacts of COVID-19 (Vol. 4) : Questionnaire Template (English). Washington, D.C.: : World Bank 2020. http://documents.worldbank.org/curated/en/567571588697439581/Questionnaire-Template

4 World Bank. COVID-19 CORE-PLUS Questionnaire: Interview Manual. Washington, D.C.: : World Bank 2020. https://documents1.worldbank.org/curated/en/431901588694657348/pdf/Interview-Manual-for-Template-Questionnaire.pdf

5 Amankwah A, Kanyanda S, Illukor J, *et al.* High Frequency Mobile Phone Surveys of Households to Assess the Impacts of COVID-19 (Vol. 3) : Guidelines on CATI Implementation (English). Washington, D.C.: : World Bank 2020. http://documents.worldbank.org/curated/en/189691588696451053/Guidelines-on-CATI-Implementation

6 World Bank. LSMS-Supported High-Frequency Phone Surveys on COVID-19. World Bank. 2020.https://www.worldbank.org/en/programs/lsms/brief/lsms-launches-high-frequency-phone-surveys-on-covid-19 (accessed 4 Aug 2021).

7 Carletto G, Kilic T. Capitalizing on the World Bank LSMS-ISA Program for High-Frequency Phone Surveys on COVID-19. Washington, D.C.: : World Bank 2020. https://thedocs.worldbank.org/en/doc/772531587652004382-0050022020/original/ProposalCapitalizingontheLSMSISAProgramforHighFrequencyPhoneSurveysonCOVID19.pdf

8 World Bank. About LSMS. LSMS. https://www.worldbank.org/en/programs/lsms/overview (accessed 4 Aug 2021).

9 Brubaker JM, Kilic T, Wollburg PR. Representativeness of Individual-Level Data in COVID-19 Phone Surveys: Findings from Sub-Saharan Africa. The World Bank 2021. https://econpapers.repec.org/paper/wbkwbrwps/9660.htm (accessed 19 May 2021).
