# Supplementary material for: Acceptance of COVID-19 vaccines in sub-Saharan Africa: evidence from six national phone surveys
Source: BMJ Open. 2021 Dec 15;11(12):e055159. doi: 10.1136/bmjopen-2021-055159 (PMC8678558; doi:10.1136/bmjopen-2021-055159)
Supplement: Author's manuscript [file bmjopen-2021-055159.draft_revisions.pdf]

BMJ Open is committed to open peer review. As part of this commitment we make the peer review history of every article we publish publicly available.

When an article is published we post the peer reviewers' comments and the authors' responses online. We also post the versions of the paper that were used during peer review. These are the versions that the peer review comments apply to.

The versions of the paper that follow are the versions that were submitted during the peer review process. They are not the versions of record or the final published versions. They should not be cited or distributed as the published version of this manuscript.

BMJ Open is an open access journal and the full, final, typeset and author-corrected version of record of the manuscript is available on our site with no access controls, subscription charges or pay-per-view fees (http://bmjopen.bmj.com).

If you have any questions on BMJ Open's open peer review process please email info.bmjopen@bmj.com

The acceptance of covid-19 vaccines in Sub-Saharan Africa: Evidence from 6 national phone surveys

Journal:	*BMJ Open*
Manuscript ID	bmjopen-2021-055159
Article Type:	Original research
Date Submitted by the Author:	04-Jul-2021

Complete List of Authors:	Kanyanda, Shelton; World Bank Group, Development Data Group<br>Markhof, Yannick; World Bank Group, Development Data Group<br>Wollburg, Philip; World Bank Group, Development Data Group<br>Zezza, Alberto; World Bank Group, Development Data Group

Keywords:	COVID-19, PUBLIC HEALTH, Health policy < HEALTH SERVICES ADMINISTRATION & MANAGEMENT

SCHOLARONE™ Manuscripts

I, the Submitting Author has the right to grant and does grant on behalf of all authors of the Work (as defined in the below author licence), an exclusive licence and/or a non-exclusive licence for contributions from authors who are: i) UK Crown employees; ii) where BMJ has agreed a CC-BY licence shall apply, and/or iii) in accordance with the terms applicable for US Federal Government officers or employees acting as part of their official duties; on a worldwide, perpetual, irrevocable, royalty-free basis to BMJ Publishing Group Ltd ("BMJ") its licensees and where the relevant Journal is co-owned by BMJ to the co-owners of the Journal, to publish the Work in this journal and any other BMJ products and to exploit all rights, as set out in our licence.

The Submitting Author accepts and understands that any supply made under these terms is made by BMJ to the Submitting Author unless you are acting as an employee on behalf of your employer or a postgraduate student of an affiliated institution which is paying any applicable article publishing charge ("APC") for Open Access articles. Where the Submitting Author wishes to make the Work available on an Open Access basis (and intends to pay the relevant APC), the terms of reuse of such Open Access shall be governed by a Creative Commons licence – details of these licences and which Creative Commons licence will apply to this Work are set out in our licence referred to above.

Other than as permitted in any relevant BMJ Author's Self Archiving Policies, I confirm this Work has not been accepted for publication elsewhere, is not being considered for publication elsewhere and does not duplicate material already published. I confirm all authors consent to publication of this Work and authorise the granting of this licence.

For peer review only

The acceptance of covid-19 vaccines in Sub-Saharan Africa: Evidence from 6 national phone surveys

Shelton Kanyanda[1], Yannick Markhof[2], Philip Wollburg[3], Alberto Zezza[4]*

[1] Consultant, World Bank, Development Data Group

[2] Consultant, World Bank, Development Data Group

[3] Economist, World Bank, Development Data Group

[4] Program Manager, World Bank, Development Data Group

* corresponding author

azezza@worldbank.org

For peer review only

Keywords: vaccines, COVID-19, Africa, vaccine hesitancy, vaccine acceptance

Word count: 3,921

Abstract

Objectives

To estimate the willingness to accept a covid-19 vaccine in six Sub-Saharan African countries and identify differences in acceptance across countries and population groups.

Design

Cross-country comparable, descriptive study based on a longitudinal survey.

Setting

Six national surveys from countries representing 38% of the Sub-Saharan African population (Burkina Faso, Ethiopia, Malawi, Mali, Nigeria, and Uganda).

Participants

Respondents of national high-frequency phone surveys (HFPS), aged 15 and older, drawn from a nationally representative sample of households.

Main outcome measures

Willingness to get vaccinated against covid-19 if an approved vaccine is provided now and for free, disaggregated by demographic attributes and socio-economic factors obtained from national household surveys. Correlates of and reasons for vaccine hesitancy.

Results

Acceptance rates in the six Sub-Saharan African countries studied are generally high, with at least four in five people willing to be vaccinated in all but one country. Vaccine acceptance ranges from nearly universal in Ethiopia (97.9%, 97.2% to 98.6%) to below what would likely be required for herd immunity in Mali (64.5%, 61.3% to 67.8%). We find little evidence for systematic differences in vaccine hesitancy by sex or age but some clusters of hesitancy in urban areas, among the better educated, and in richer households. Safety concerns about the vaccine in general and its side effects specifically emerge as the primary reservations toward a covid-19 vaccine across countries.

Conclusions

Our findings suggest that limited supply, not inadequate demand, is likely to present the key bottleneck to reaching high covid-19 vaccine coverage in Sub-Saharan Africa. To turn intent into effective demand, targeted communication campaigns bolstering confidence in the safety of approved vaccines and reducing concerns about side effects will be crucial to safeguard the swift progression of vaccine rollout in one of the world's poorest regions.

Article Summary

Strengths and limitations of this study

- Much of the current debate on vaccination campaigns in low-income countries in general and Sub Saharan Africa (SSA) in particular focuses on supply-chain and financial factors, yet there is a dearth of robust, comparable evidence on covid-19 vaccine hesitancy in these countries.
- We use cross-country comparable data from six national high-frequency phone surveys in SSA to fill this crucial knowledge gap
- Our sample is drawn from large, nationally representative sampling frames that provide a rich set of demographic and socio-economic characteristics by which we disaggregate our analysis
- Through a set of re-calibrated survey weights, our analysis adjusts for the selection biases common in other remote surveys, yet, some bias may persist
- Attitudes toward vaccines are malleable and may change over time

Introduction

As vaccination campaigns in high-income countries are accelerating, large swathes of the global population living in low- and middle-income countries remain severely exposed to the novel coronavirus disease (covid-19).[1] Sub-Saharan Africa is home to 433 million people living below the international absolute poverty line (about two thirds of the global poor population).[2] For these populations, non-pharmaceutical interventions to curb the spread of the disease impinged on already precarious livelihoods.[4] Furthermore, the region is characterized by high prevalence of comorbidities, and public health systems that are ill equipped to stave off the burden of mass infection.[1,3,4] Reaching large-scale vaccination coverage in Sub-Saharan Africa (SSA) is thus pivotal in the global effort to halt the spread of the disease and limit its toll on lives and livelihoods.[5–7]

Recent calls to action by international stakeholders and a growing body of scholarly research focus on supply-chain and financial factors to safeguard sufficient vaccine availability in SSA. The COVAX initiative aims to provide doses sufficient to vaccinate up to 20% of the population in the region, far below the target population required for a coverage of 60%, or 321.5 million individuals.[8,9] Still, this would leave an estimated financing gap of over $10 billion in SSA.[10] A June 2021 resolution from the G7 pledging one billion covid-19-vaccine doses for low-income countries offers hope for those supply gaps being filled. While these considerations concern the availability of vaccines, another key factor for the success of vaccination campaigns in SSA is the willingness to be vaccinated within the population. Yet, there is a dearth of large-scale evidence on vaccine acceptance in low income countries in general and SSA in particular.

Based on a cross-country comparable sample with national scope from six Sub-Saharan African countries, we fill this knowledge gap by providing estimates of vaccine acceptance for a population representative of around 416 million people, 38% of the population of Sub-Saharan Africa.[11] Drawing on the high frequency phone surveys (HFPS) based on pre-pandemic sampling frames from nationally representative, face-to-face household surveys supported by the World Bank's Living Standard Measurement Study – Integrated Survey on Agriculture (LSMS-ISA), we are able to link covid-19 vaccine acceptance rates to a rich set of demographic and socio-economic characteristics. Along with recalibrated sampling weights, this allows our study to provide robust insights into the likelihood of the current efforts to ensure sufficient supply of vaccination doses to also meet adequate demand in SSA, identify clusters of hesitancy, and contribute to a swift rollout of vaccination campaigns where they will be needed most.

Methods

Data and survey instrument

We use data from the national longitudinal high-frequency phone surveys on covid-19 (HFPS) conducted in Burkina Faso, Ethiopia, Malawi, Mali, Nigeria, and Uganda. Survey implementation was led by the respective national statistical agencies, the Burkina Faso National Institute of Statistics and Demography; Laterite Ethiopia in collaboration with the Central Statistical Agency of Ethiopia; Malawi National Statistical Office; Mali National Institute of Statistics; Nigeria

For peer review only

Bureau of Statistics; and Uganda Bureau of Statistics; and supported by the World Bank Living Standards Measurement Study and the Poverty and Equity Global Practice. The HFPS on covid-19 have been implemented monthly since May 2020, aiming to gauge the impact of the covid-19 pandemic on individual and household attitudes, socioeconomic and health outcomes.[12]

The HFPS rounds in which vaccine hesitancy was covered in a cross-country comparable fashion were conducted in September 2020 (Ethiopia, round 6), October-November 2020 (Malawi, round 5; Mali, round 5; and Nigeria, round 6) and December 2020 (Burkina Faso, round 5; and Uganda, round 4). The primary survey questions of interest, posed to each phone survey respondent, was: '*If an approved vaccine to prevent coronavirus was available right now at no cost, would you agree to be vaccinated?'* with response options '*yes*', '*no*', and '*not sure*';. If respondents answered '*no*' or *'not sure'*, this was followed up by a question about possible reasons for refusing to be vaccinated: '*What are the reasons you would not agree to be vaccinated?',* with response options:

(1) 'I don't think it will be safe'
(2) 'I am worried about the side effects'
(3) 'It is against my religion'
(4) 'I am not at risk of contracting covid-19'
(5) 'I don't think it will work'
(6) 'I am against vaccine in general'
(7) Other

We are interested in the association between willingness to be vaccinated and a standard set of demographic and socioeconomic variables, such as gender, age, income, and education. These are available for respondents either from the HFPS directly or from pre-covid-19 face-to-face (FtF) surveys, which served as the sampling frames for the HFPS. We further draw on multiple rounds of the HFPS to create variables on respondents' attitudes towards the covid-19 emergency and how it has affected them and their families. These include willingness to be tested for covid-19, rating of government response, and whether households received any government assistance during the pandemic. The data as well as survey instruments and basic information documents are available publicly on the World Bank Microdata Library under the High-Frequency Phone Survey collection.[13]

Sampling and sample representativeness

The samples for the HFPS were drawn from mobile phone numbers recorded during data collection for nationally representative face-to-face (FtF) household surveys implemented prior to the covid-19 pandemic with support from the World Bank Living Standards Measurement Study – Integrated Surveys on Agriculture (LSMS-ISA) program. Specifically, the Burkina Faso Enquête harmonisée sur les conditions de vie des ménages 2018/19, Ethiopia Socioeconomic Survey 2018/19, Malawi Integrated Household Panel Survey 2019, Mali Enquête harmonisée sur les conditions de vie des ménages 2018/19, Nigeria General Household Survey – Panel 2018/19, and Uganda National Panel Survey 2019/20. At least one phone number was recorded for all households with access to a phone, including through a contact person outside of the household, such as a friend or neighbor. For the HFPS, Ethiopia, Malawi, and Uganda attempted to contact all households with available

For peer review only

phone numbers, while Burkina Faso, Nigeria and Mali selected a random sub-sample of phone numbers to call (Table A.1).

In the absence of universal access to a mobile phone, the HFPS households are likely to be selected samples of the nationally representative FtF survey samples of households.[14] The publicly available HFPS datasets therefore come with recalibrated household survey weights to counteract potential selection biases. The recalibration model takes advantage of the rich information on households with and without access to a phone from the pre-covid-19 FtF surveys [15,16] and follows a methodology proposed in a reference methodological paper on this subject.[17] The effectiveness of this weight recalibration in overcoming selection biases has been documented in HFPS data from four of the six countries we study, such that household-level estimates based on the HFPS data can be broadly considered representative at the national level.[14]

Further, the HFPS survey questions on vaccinations were asked only to the household's main respondent, who had to be 15 or older, as it is impractical to interview all individuals in a household in a phone survey. The selection of main respondents was not randomized so that the group of respondents is likely not representative of the general population of adults at the individual level. Rather, respondents tend to be household heads or their spouses, better educated and slightly older, as a recent study documents.[18] An additional recalibration of survey weights for individual-level analysis improves representativeness, but cannot overcome respondent selection biases in all variables and increases the variance of the estimates.[18]

Considering these potential shortfalls in the representativeness of our data, we present our main results first with the standard HFPS household weights and then, as a robustness and sensitivity check for potential respondent selection biases, with the recalibrated individual-level weights. A summary of unweighted individual characteristics of FtF adults vis-à-vis HFPS adults is presented in Table A.3.

Statistical analysis

The statistical analysis proceeds in several steps. First, we estimate the weighted mean of willingness to get vaccinated by country, and within countries by sex of respondent, residence (urban and rural) and income quintile, as well as reasons for vaccine hesitancy, using the recalibrated household weights. Second, we explore how individual and household characteristics, such as education and expenditure, correlate with the willingness to get vaccinated in a set of multivariate logit regressions, again using household weights. To assess how much the differences in the attributes of respondents vis-à-vis the general adult population may affect the representativeness of our results, we assess the sensitivity of the results to using individual-level recalibrated weights instead of the public-use household weights (see Discussion).[18]

Patient and public involvement

This research did not involve consultation with patients or the public.

For peer review only

Results

Descriptive results

Overall, we find high levels of willingness to be vaccinated: Acceptance is estimated to be nearly universal in Ethiopia at 97.9% (95% confidence interval 97.2% to 98.6%) and very high in Nigeria (86.2%, 83.9% to 88.5%), Uganda (84.5%, 82.2% to 86.8%), Malawi (82.7%, 80.0% to 85.4%), and Burkina Faso (79.5%, 76.9% to 82.1%). In these countries at least four in five respondents would agree to be vaccinated if an approved vaccine was made available for free to them (Figure 1, Panel A). Acceptance is somewhat lower only in Mali where less than two thirds of respondents (64.5%, 61.3% to 67.8%) reported their willingness to be vaccinated. Notably, Mali is also the only country in which a non-negligible share (12.4%) is uncertain about their answer, a fact that accounts for the majority of the lower acceptance rates in Mali. For the following analysis, we focus on acceptance rates and do not distinguish between respondents rejecting to be vaccinated and those that are not sure. Pooling the data from all countries and weighting them by their respective popualtion sizes yields an overall mean acceptance rate of 87.6% (86.4% to 88.8%) across the six countries (Table A.2).

In Panel B of Figure 1, we disaggregate acceptance rates by respondent sex. Except for Nigeria where acceptance is statistically significantly higher among male than female respondents (90.1%, 87.6% to 92.6% and 75.7%, 70.4% to 80.9%, respectively) and in Ethiopia where there is a small, yet statistically significant difference (98.7%, 97.9% to 99.4% and 95.8%, 94.1% to 97.5%, respectively), we do not find answers to differ between men and women. Similarly, Panel C reports differences in willingness to be vaccinated against covid-19 between rural and urban areas with higher acceptance in rural areas in Burkina Faso, Ethiopia, and Malawi at the 95% confidence level. One distinct feature of our data is the ability to tap into the rich pre-covid-19 baseline data from the face-to-face household surveys that served as sampling frames. This way, we are able to determine respondent household's position in the national expenditure distribution and disaggregated acceptance rates by expenditure quintile (Panel D). We generally find a downward sloping pattern in which acceptance is higher among poorer households and lowest among the richest households. This pattern is particularly evident in Burkina Faso but also noteworthy in Uganda and Nigeria. Conversely, acceptance is high throughout all expenditure quintiles in Ethiopia and not significantly lower for the richest two quintiles in Mali.

For those respondents who were hesitant to be vaccinated, the survey asked to provide a reason. From Figure 2, it is evident that safety concerns were paramount ranging from 53.7% (36.1% to 71.3%) of those reporting they would refuse to be vaccinated in Burkina Faso to 42.4% (33.8% to 50.9%) in Malawi, despite the wording of the questions making it explicit that the vaccine would be officially approved. Other notable reasons include worries about the potential side effects of the vaccine (between 55% (46.9% to 63.0%) in Uganda and 12.8% (9.1% to 16.5%) in Mali) and the belief not to be at risk of contracting the virus (34.2% (25.2% to 43.3%) in Nigeria to 0.1% (0% to 2.1%) in Burkina Faso).

For peer review only

Correlates of hesitancy

Exploiting the richness of our data, we can explore correlational patterns between willingness to be vaccinated for covid-19 and a large set of respondent and household characteristics. Table 1 confirms in a multivariate setting the patterns observed graphically earlier. In general, men tend to be more willing to take the vaccine, though we only detect a statistically significant effect in Nigeria. Respondents in urban areas are more skeptical with significant differences in Burkina Faso, Ethiopia, and Malawi.

An association we observe across countries is between education and vaccine hesitancy. In Burkina Faso, Ethiopia, Malawi, and Nigeria, those with more years of education are significantly less willing to be vaccinated while coefficients signs point in the same direction in Mali and Uganda but are not significant. Lastly, we do not find a pattern according to respondents' age and a mixed picture according to the role of the respondent in the household. Here, household heads which constitute the majority of our sample (see Table A.3) are more willing to be vaccinated in Ethiopia, more hesitant in Mali, and not significantly different from other household members in the remaining four cases.

A pattern we observe in several countries is a higher hesitancy toward the vaccine in richer households compared to the poorest expenditure quintile, with coefficients significant in Mali and Uganda for the richest two quintiles, for the top quintile in Burkina Faso and Nigeria, and the third and fourth quintiles in Malawi.

The data further show a strong association between vaccine hesitancy and the willingness to be tested for covid-19 suggesting similar underlying reasons for (or against) testing and getting vaccinated. Another hypothesis we explore is whether there is an association between the receipt of some assistance during the pandemic, e.g. through one of the large-scale social protection programs launched to combat the fallout of the pandemic, and willingness to receive a free, approved vaccine against covid-19. While coefficient signs mostly point to a positive association, the effect is not statistically significantly different from zero despite all countries in our sample launching some social protection response to the crisis.[19]

A last hypothesis we pursue concerns the relationship between trust in and satisfaction with the government and willingness to be vaccinated. For example, more skeptical individuals toward the government or those dissatisfied with its crisis management may be more reluctant to accept a state-provided vaccine.[20,21] We only have data on trust in the government's crisis management in Malawi and satisfaction in Mali and Nigeria but find our hypotheses confirmed in bivariate logistic regressions (Table A.6). However, after controlling for all other factors in Table 1, coefficients are no longer significant.

For peer review only

Table 1: Correlates of hesitancy.

Correlates of hesitancy - marginal effects from multivariate logit regressions

	(1)	(2)	(3)	(4)	(5)	(6)
VARIABLES	Burkina Faso	Ethiopia	Malawi	Mali	Nigeria	Uganda
Respondent is male	-0.0388	0.00913	0.0419	0.0522	0.0932***	0.0179
	(0.0340)	(0.00569)	(0.0311)	(0.0413)	(0.0268)	(0.0224)
Lives in urban area	-0.0878***	-0.0191**	-0.0880***	0.0237	-0.0151	0.0168
	(0.0294)	(0.00858)	(0.0289)	(0.0286)	(0.0222)	(0.0212)
Years of education	-0.00610**	-0.00137*	-0.0135***	-0.00452	-0.00680**	-0.00164
	(0.00252)	(0.000734)	(0.00330)	(0.00288)	(0.00286)	(0.00283)
Age group 30-60 yrs	-0.0166	0.00494	0.0152	0.0297	0.0436	-0.0274
	(0.0365)	(0.00838)	(0.0312)	(0.0458)	(0.0366)	(0.0269)
Age group 60+ yrs	0.0446	0.00700	-0.0140	-0.0273	0.0499	0.0179
	(0.0435)	(0.0141)	(0.0505)	(0.0584)	(0.0461)	(0.0341)
Household head	0.0558	0.0171**	-0.00894	-0.0886**	-0.0266	-0.00563
	(0.0376)	(0.00776)	(0.0381)	(0.0450)	(0.0325)	(0.0242)
Household Size	0.00310	0.000705	0.0103*	-0.00536	0.00286	0.00631
	(0.00378)	(0.00152)	(0.00557)	(0.00358)	(0.00580)	(0.00476)
Expenditure, 2nd quintile	0.0230	0.00677	0.0198	0.0307	-0.0677	-0.0302
	(0.0426)	(0.0102)	(0.0437)	(0.0441)	(0.0564)	(0.0319)
Expenditure, 3rd quintile	-0.0568	-0.0216	-0.0953*	0.0166	0.00195	-0.0500
	(0.0430)	(0.0195)	(0.0516)	(0.0431)	(0.0427)	(0.0308)
Expenditure, 4th quintile	-0.0357	-0.00381	-0.116**	-0.0932*	-0.0623	-0.107***
	(0.0442)	(0.0103)	(0.0473)	(0.0501)	(0.0458)	(0.0340)
Expenditure, 5th (richest) quintile	-0.164***	-0.0111	-0.0106	-0.135**	-0.0696*	-0.0754**
	(0.0534)	(0.0108)	(0.0457)	(0.0536)	(0.0421)	(0.0346)
Willing to be tested for covid-19	0.208***	0.0619***	0.253***	0.461***	0.221***	0.340***
	(0.0313)	(0.00889)	(0.0389)	(0.0172)	(0.0221)	(0.0254)
HH received assistance during covid-19	0.0490	0.00607	-0.00457	-0.00728	0.0152	0.0307
	(0.0470)	(0.00886)	(0.0322)	(0.0618)	(0.0220)	(0.0194)
Gov't not trustworthy			-0.0455			
			(0.0291)			
Satisfied with gov't response				0.0354	0.0279	
				(0.0450)	(0.0202)	
Observations	1,742	2,654	1,542	1,591	1,703	2,106
Pseudo R2	0.183	0.308	0.137	0.265	0.218	0.233

Robust standard errors in parentheses. *** p<0.01, ** p<0.05, * p<0.1. Weighted logit regressions with willingness to take a free, approved vaccine as dependent variable.

For peer review only

Discussion

Principal findings

Our study uses cross-country comparable data from the national longitudinal high frequency phone surveys on covid-19 (HFPS) in six Sub-Saharan African countries to estimate acceptance rates for an approved, free covid-19 vaccine. By linking phone survey data to the nationally representative, large-scale, face-to-face household surveys that served as sampling frames for the HFPS, we provide robust estimates of the willingness to be vaccinated against covid-19 across a diverse set of demographics and respondent and household characteristics. Our headline results indicate high acceptance rates with at least four in five respondents signaling their willingness to be vaccinated in all but one of the countries studied. There is cross-country variation, with willingness to be vaccinated ranging from 64.5% in Mali (61.3% to 67.8%), where a further 12.4% are yet undecided, to near universal acceptance in Ethiopia (97.9%, 97.2% to 98.6%). We also find little evidence for systematic differences in vaccine hesitancy across gender or age but some notable clusters of hesitancy in urban areas, among those better educated, and in richer households. Across countries, safety concerns about the vaccine in general and its side effects specifically emerged as the primary reservations toward a covid-19 vaccine.

Strengths and comparison with other studies

In relation to the existing literature, our study has several advantages in the domains of coverage and sample selection, richness of the data, and analytical depth. In terms of coverage, our study focuses on a region with yet scant evidence on willingness to be vaccinated against covid-19. Importantly, the data we assemble are cross-country comparable and without exception based on large, nationally representative sampling frames from pre-covid, face-to-face household surveys. This allows us to calibrate survey weights that adjust for the coverage and non-response biases that plague other studies at a time where regular face-to-face data collection nearly came to a complete halt.[22–24] Furthermore, our study is unique in that the pre-existing survey data from which our samples are drawn allow us to tap into a rich set of baseline individual and household characteristics. This facilitates a more rigorous analysis and disaggregation of acceptance rates, including, for instance, households' position in the national (pre-crisis) expenditure distribution, than has been possible previously. Our study thus stands out as a fully cross-country comparable, multivariate, and inferential analysis of vaccine hesitancy on the African continent.

Compared to our study, previous studies that analyze covid-19 vaccine acceptance rates and the reasons for refusal predominantly (i) focus on middle- and high-income countries, (ii) represent single-country or not cross-country comparable samples, (iii) study particular subpopulations such as university students, healthcare workers, or participants of unrelated pre-covid studies, (iv) rely on non-random sampling, or (v) can only draw on a small set of demographics and characteristics to disaggregate their analysis.[18,25–35]

A cross-country study of 19 countries with samples obtained from commercial online panel providers found 71.5% of respondents willing to be vaccinated against covid-19 with a rate of 65.2% in Nigeria as the only low-income country.[36] Similarly, a literature review of 31 studies

For peer review only - http://bmjopen.bmj.com/site/about/guidelines.xhtml

covering 33 countries found covid-19 vaccine acceptance rates at or above 70% among studies focusing on the general population but also large regional and intra-regional differences and a dearth of evidence particularly from Sub-Saharan Africa.[37]

Few studies explicitly focus on acceptance rates in the poorest countries. Assembling an amalgamation of data samples with different sources, sampling methodologies, and coverage, one study finds generally high acceptance rates in ten LMICs in Asia, Africa, and South America.[38] Among countries also covered in our study, acceptance rates are lower than what we find in Burkina Faso (66.5%, national phone sample obtained by random digit dialing) and Nigeria (76.2%, random sample of residents of one state from telephone list), close in urban Uganda (76.5%, random sample of households in Kampala), and very similar in rural Uganda (85.8%, non-random sample of women in 13 districts).[38] However, in those studies the ability to correct for various sample selection biases is likely limited in the absence of baseline nationally representative sampling frames. Another study in 15 African countries, including Burkina Faso, Malawi, Nigeria, Uganda (all face-to-face interviewing), and Ethiopia (telephone survey) found estimates for the countries also covered in our study mostly in the vicinity of our weighted figures (Ethiopia 94%, Uganda 87.5%, Burkina Faso 86%, Malawi 82.7%, Nigeria 76%).[39,40] Notably, average sample sizes are roughly half of our study's and there is a lack of analytical detail impeding a robust inference of cross-country and -demographic findings. Lastly, our results contrast with a recent Afrobarometer study based on in-person interviews in five West African countries, none of which are also included in our sample. In relation to our study, the study's analysis is purely descriptive and reports low acceptance rates of 40% on average.[21] Similar to what we find, the study cites trust in vaccine safety as the key driver of hesitancy, a result that is further corroborated in the literature.[21,28,36,38,40] Furthermore, the small systematic differences we observe according to gender and age are in line with most previous findings in low-income countries as is a tendency for higher acceptance in rural areas.[21,36,38,40] As with overall acceptance rates, our finding of higher hesitancy among the more educated is in line with two of the studies covering Sub-Saharan Africa [38,39] while another study finds mixed evidence.[21] No other study we are aware of across LMICs (and in fact few across countries of any income classification) assess vaccine acceptance according to economic status in a manner comparable with the expenditure data we can access from the pre-covid-19 sampling frames.

Limitations of this study

This study uses data from high-frequency phone surveys (HFPS) with national coverage along with sampling weights specifically recalibrated for the phone surveys to be nationally representative. The weights were shown to be effective at achieving nationally representative estimates at the household level.[14] However, willingness to be vaccinated is primarily not a household-level attribute but an individual decision, though it is reasonable to expect considerable intra-household correlation in attitudes towards vaccination. Phone survey respondents in our data are not specifically selected to be representative of all individuals and that may limit the population-level representativeness of the results we report. Recalibration of survey weights at the individual level can partially but not fully address this concern.[18] To gauge how sensitive our results are to respondent selection biases in individual-level data, we compare the estimates with

For peer review only

household-level weights to the same estimates with individual-level weights. In this test, large deviations between those two sets of estimates would indicate that selection biases affect our results in a fundamental way. However, we find only limited change in the estimates, regardless of which weight is used: in over three quarters of cases, deviations do not exceed two percentage points, including the headline findings on willingness to be vaccinated in all study countries, and we find only three instances where the differences exceed five points (Variable 'Q5' 5.8% in Burkina Faso; 'Q4' 6.2% in Mali; 'Q3' 6.0% in Nigeria; Table A.4). The results from the multivariate logit regression model are also robust to the use of household or individual-level weights. This is true especially of the cross-country findings for urban and richer households and willingness to get tested for covid-19: the point estimates tend to be slightly larger when using individual-level compared to household-level weights but they are of comparable magnitude and in most cases retain statistical significance (Table A.5). In contrast, the findings on education have similar point estimates whether we use household or individual-level weights, but with individual-level weights they retain statistical significance only in Malawi. This is likely because individual-level weight recalibration increases the variance of the estimates. All in all, we take this set of tests to indicate the robustness of our results to concerns around respondent selection.

Another potential limitation is the possible malleability of attitudes towards vaccinations, which may have changed, and continue to change, in light of the development of various vaccines and the relative success these appear to have in stemming the pandemic in other parts of the world. Future research is needed to determine evolving attitudes in Africa towards being vaccinated and their interactions with vaccine supply and availability.

Conclusions and policy implications

Our headline results of high vaccine acceptance in a cross-country comparable sample of six Sub-Saharan African countries suggests that limited supply, not inadequate demand, is likely to present the key bottleneck to reaching high covid-19 vaccine coverage in the region. As willingness to be vaccinated does not automatically translate into vaccine-seeking behavior, public authorities need to turn intent into effective demand as vaccine rollout progresses.[41] For this, our study identifies some indicative pockets of hesitancy, particularly in Mali, urban Burkina Faso and Malawi, among women in Nigeria, and for richer households and those with more education. As many of these population groups are easier to reach early in the vaccine rollout process, yet are also better reachable through targeted communication, campaigns that raise acceptance for a covid-19 vaccine in these clusters will be key. These should focus on resolving concerns about side effects and bolster confidence in the safety of approved covid-19 vaccines in order to reach mass coverage and end the pandemic swiftly and everywhere.

For peer review only

Author contributions

All authors had full access to all of the data in the study and take responsibility for the integrity of the data and the accuracy of the data analysis. All authors were responsible for its conception and design. YM, PW, and SK were responsible for the preparation and analysis of the data and all authors contributed to their interpretation. YM and PW drafted the manuscript and AZ made critical revision of the manuscript for important intellectual content. SK and AZ conceptualized an early version. AZ is the guarantor. The corresponding author attests that all listed authors meet authorship criteria and that no others meeting criteria have been omitted.

Funding

Funding for the analysis comes from the World Bank Multi-Donor Trust Fund for Integrated Household and Agricultural Surveys in Low and Middle-Income Countries (TF072496). The funders had no role in study design, data collection, analysis, interpretation of the data, decision to publish, or preparation of the manuscript.

Competing interests

All authors have completed the ICMJE uniform disclosure form at www.icmje.org/coi_disclosure.pdf and declare: no support from any organization for the submitted work; no financial relationships with any organizations that might have an interest in the submitted work in the previous three years; no other relationships or activities that could appear to have influenced the submitted work.

Ethics approval

Each phone survey was implemented by the respective national statistical office (NSO), except for Ethiopia where a private firm was the implementing agency. In Burkina Faso, Malawi, Mali, Nigeria, and Uganda, the NSO conducts the survey as the sole official statistical authority in the country and in accordance with the respective National Statistical Act, which exempts the NSO from institutional ethics approvals. Informed consent was received from all survey respondents in each country. The World Bank does not require institutional ethics approval for household surveys that are partly or fully financed by the World Bank, including the national phone surveys in Burkina Faso, Ethiopia, Malawi, Mali, Nigeria, and Uganda that inform our research.

Data Sharing

The data are available from the World Bank's Microdata Library, High Frequency Phone Survey Catalog (https://microdata.worldbank.org/index.php/catalog/hfps) as well as LSMS catalog (https://microdata.worldbank.org/index.php/catalog/lsms).

Transparency Statement

The authors affirm that the manuscript is an honest, accurate, and transparent account of the study being reported; that no important aspects of the study have been omitted; and that any discrepancies from the study as planned have been explained.

Dissemination to participants and related patient and public communities:

Our research uses data from a large, anonymized sample of participants and there are thus no plans to disseminate the research to specific participants or patients beyond publishing it.

For peer review only

Licensing

The Corresponding Author is aware that the World Bank external publishing department has recently agreed to grant to BMJ Publishing Group Limited a worldwide, non-exclusive license to publish an article produced by World Bank staff and consultants, and is committed to facilitate the conclusion of a similar agreement should this manuscript be accepted for publication.

References

1 Schellekens P, Indermit G. COVID-19 is a developing country pandemic. Future Development. 2021.https://www.brookings.edu/blog/future-development/2021/05/27/covid-19-is-a-developing-country-pandemic/ (accessed 10 Jun 2021).

2 World Bank. *Poverty and Shared Prosperity 2020 : Reversals of Fortune*. Washington, DC: World Bank 2020. doi:10.1596/978-1-4648-1602-4

3 Rice BL, Annapragada A, Baker RE, *et al.* Variation in SARS-CoV-2 outbreaks across sub-Saharan Africa. *Nat Med* 2021;**27**:447–53. doi:10.1038/s41591-021-01234-8

4 Josephson A, Kilic T, Michler JD. Socioeconomic impacts of COVID-19 in low-income countries. *Nat Hum Behav* Published Online First: 30 March 2021. doi:10.1038/s41562-021-01096-7

5 Britton T, Ball F, Trapman P. A mathematical model reveals the influence of population heterogeneity on herd immunity to SARS-CoV-2. *Science* 2020;**369**:846–9. doi:10.1126/science.abc6810

6 Aschwanden C. Five reasons why COVID herd immunity is probably impossible. *Nature* 2021;**591**:520–2. doi:10.1038/d41586-021-00728-2

7 Schellekens P. For greater vaccine equity, first fix these misconceptions. Future Development. 2021.https://www.brookings.edu/blog/future-development/2021/04/02/for-greater-vaccine-equity-first-fix-these-misconceptions/ (accessed 10 Jun 2021).

8 Berkley S. COVAX explained. #Vaccineswork. 2020.https://www.gavi.org/vaccineswork/covax-explained (accessed 19 May 2021).

9 Wang W, Wu Q, Yang J, *et al.* Global, regional, and national estimates of target population sizes for covid-19 vaccination: descriptive study. *BMJ* 2020;:m4704. doi:10.1136/bmj.m4704

10 Yoo KJ, De Francisco Serpa N, Gordillo-Tobar A. Calculating Sub-Saharan Africa's COVID vaccination financing gap. Investing in Health. 2021.https://blogs.worldbank.org/health/calculating-sub-saharan-africas-covid-vaccination-financing-gap (accessed 19 May 2021).

11 World Bank. Population, total. World Development Indicators. 2019.https://data.worldbank.org/indicator/SP.POP.TOTL (accessed 15 Jun 2021).

12 Fu H, Sanchez-Paramo C. High-frequency monitoring of COVID-19 impacts. 2020.https://blogs.worldbank.org/opendata/high-frequency-monitoring-covid-19-impacts (accessed 27 Oct 2020).

For peer review only

13 High-Frequency Phone Surveys. World Bank Microdata Library. https://microdata.worldbank.org/index.php/catalog/hfps (accessed 10 May 2021).

14 Ambel A, McGee K, Tsegay A. Reducing Bias in Phone Survey Samples. Effectiveness of Reweighting Techniques using Face-to-Face Surveys as Frames. Forthcoming.

15 Himelein K, Eckman S, Kastelic J, *et al.* High Frequency Mobile Phone Surveys of Households to Assess the Impacts of COVID-19. Guidelines on Sampling Design. Washington D.C.: : World Bank 2020. http://documents.worldbank.org/curated/en/742581588695955271/Guidelines-on-Sampling-Design

16 Josephson A, Kilic T, Michler JD. Socioeconomic Impacts of COVID-19 in Four African Countries. Washington D.C.: : The World Bank 2020.

17 Himelein K. Weight Calculations for Panel Surveys with Subsampling and Split-off Tracking. *Statistics and Public Policy* 2014;**1**:40–5. doi:10.1080/2330443X.2013.856170

18 Brubaker JM, Kilic T, Wollburg PR. Representativeness of Individual-Level Data in COVID-19 Phone Surveys: Findings from Sub-Saharan Africa. The World Bank 2021. https://econpapers.repec.org/paper/wbkwbrwps/9660.htm (accessed 19 May 2021).

19 Gentilini U, Almenfi M, Blomquist J, *et al.* Social Protection and Jobs Responses to COVID-19: A Real-Time Review of Country Measures (May 14, 2021). Washington D.C.: : World Bank 2021.

20 Stoop N, Hirvonen K, Maystadt J-F. Institutional mistrust and child vaccination coverage in Africa. *BMJ Global Health* 2021;**6**:e004595. doi:10.1136/bmjgh-2020-004595

21 Seydou A. Who wants COVID-19 vaccination? In 5 West African countries, hesitancy is high, trust low. Afrobarometer 2021. https://bit.ly/3hz1wZg

22 World Bank, UNDESA. Monitoring the State of Statistical Operations under the COVID-19 Pandemic : Highlights from the Third Round of a Global COVID-19 Survey of National Statistical Offices (NSOs). Washington, D.C.: : The World Bank 2020. http://documents.worldbank.org/curated/en/833751608029512958/Monitoring-the-State-of-Statistical-Operations-under-the-COVID-19-Pandemic-Highlights-from-the-Third-Round-of-a-Global-COVID-19-Survey-of-National-Statistical-Offices-NSOs

23 World Bank, UNDESA. Monitoring the State of Statistical Operations under the COVID-19 Pandemic. Washington, D.C.: : World Bank 2020. https://documents.worldbank.org/en/publication/documents-reports/documentdetail/338481591648922978/monitoring-the-state-of-statistical-operations-under-the-covid-19-pandemic

24 World Bank, UNDESA. Monitoring the State of Statistical Operations under the COVID-19 Pandemic : Highlights from the Second Round of a Global COVID-19 Survey of National Statistical Offices (NSOs). Washington, D.C.: : World Bank 2020. https://documents1.worldbank.org/curated/en/297221597442670485/pdf/Monitoring-the-State-of-Statistical-Operations-under-the-COVID-19-Pandemic-Highlights-from-the-Second-Round-of-a-Global-COVID-19-Survey-of-National-Statistical-Offices.pdf

For peer review only

25 Barello S, Nania T, Dellafiore F, *et al.* 'Vaccine hesitancy' among university students in Italy during the COVID-19 pandemic. *Eur J Epidemiol* 2020;**35**:781–3. doi:10.1007/s10654-020-00670-z

26 Malik AA, McFadden SM, Elharake J, *et al.* Determinants of COVID-19 vaccine acceptance in the US. *EClinicalMedicine* 2020;**26**:100495. doi:10.1016/j.eclinm.2020.100495

27 Al-Mohaithef M, Padhi BK. Determinants of COVID-19 Vaccine Acceptance in Saudi Arabia: A Web-Based National Survey. *JMDH* 2020;**Volume 13**:1657–63. doi:10.2147/JMDH.S276771

28 Skjefte M, Ngirbabul M, Akeju O, *et al.* COVID-19 vaccine acceptance among pregnant women and mothers of young children: results of a survey in 16 countries. *Eur J Epidemiol* 2021;**36**:197–211. doi:10.1007/s10654-021-00728-6

29 Alqudeimat Y, Alenezi D, AlHajri B, *et al.* Acceptance of a COVID-19 Vaccine and its Related Determinants among the General Adult Population in Kuwait. *Med Princ Pract* Published Online First: 22 January 2021. doi:10.1159/000514636

30 Schwarzinger M, Watson V, Arwidson P, *et al.* COVID-19 vaccine hesitancy in a representative working-age population in France: a survey experiment based on vaccine characteristics. *The Lancet Public Health* 2021;**6**:e210–21. doi:10.1016/S2468-2667(21)00012-8

31 Benham B, JH Bloomberg School of Public Health. COVID-19 Vaccine Acceptance Falling Globally and in the U.S., Survey Finds. Johns Hopkins Bloomberg School of Public Health. 2021.https://www.jhsph.edu/news/news-releases/2021/covid-19-vaccine-acceptance-falling-globally-and-in-the-us-survey-finds.html (accessed 26 May 2021).

32 El-Elimat T, AbuAlSamen MM, Almomani BA, *et al.* Acceptance and attitudes toward COVID-19 vaccines: A cross-sectional study from Jordan. *PLoS ONE* 2021;**16**:e0250555. doi:10.1371/journal.pone.0250555

33 Brenan M. Willingness to Get COVID-19 Vaccine Ticks Up to 63% in U.S. Gallup. 2020.https://news.gallup.com/poll/327425/willingness-covid-vaccine-ticks.aspx (accessed 10 Jun 2021).

34 Funk C, Tyson A. Intent to Get a COVID-19 Vaccine Rises to 60% as Confidence in Research and Development Process Increases. Pew Research Center 2020.

35 Neumann-Böhme S, Varghese NE, Sabat I, *et al.* Once we have it, will we use it? A European survey on willingness to be vaccinated against COVID-19. *The European Journal of Health Economics* 2020;**21**:977–82. doi:10.1007/s10198-020-01208-6

36 Lazarus JV, Ratzan SC, Palayew A, *et al.* A global survey of potential acceptance of a COVID-19 vaccine. *Nat Med* 2021;**27**:225–8. doi:10.1038/s41591-020-1124-9

37 Sallam M. COVID-19 Vaccine Hesitancy Worldwide: A Concise Systematic Review of Vaccine Acceptance Rates. *Vaccines* 2021;**9**. doi:10.3390/vaccines9020160

38 Solís Arce JS, Warren SS, Meriggi NF, *et al.* COVID-19 Vaccine Acceptance and Hesitancy in Low and Middle Income Countries, and Implications for Messaging. Public and Global Health 2021. doi:10.1101/2021.03.11.21253419

For peer review only

39 Africa CDC. COVID-19 Vaccine Perceptions: A 15 country study. 2021. https://africacdc.org/download/covid-19-vaccine-perceptions-a-15-country-study/ (accessed 18 May 2021).

40 Kihara AB. Vaccination against COVID-19 in Africa. *Int J Gynecol Obstet* 2021;**153**:186–7. doi:10.1002/ijgo.13646

41 MacDonald NE. Vaccine hesitancy: Definition, scope and determinants. *Vaccine* 2015;**33**:4161–4. doi:https://doi.org/10.1016/j.vaccine.2015.04.036

For peer review only

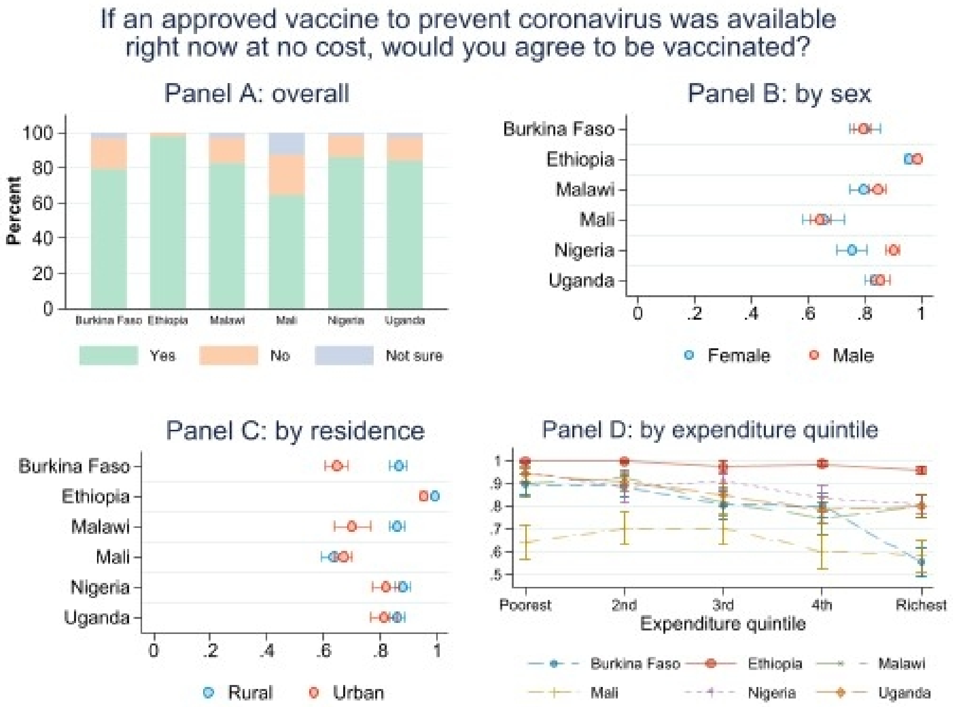

Figure 1: Vaccine hesitancy overall, by sex, by residence, and by expenditure quintile

139x101mm (96 x 96 DPI)

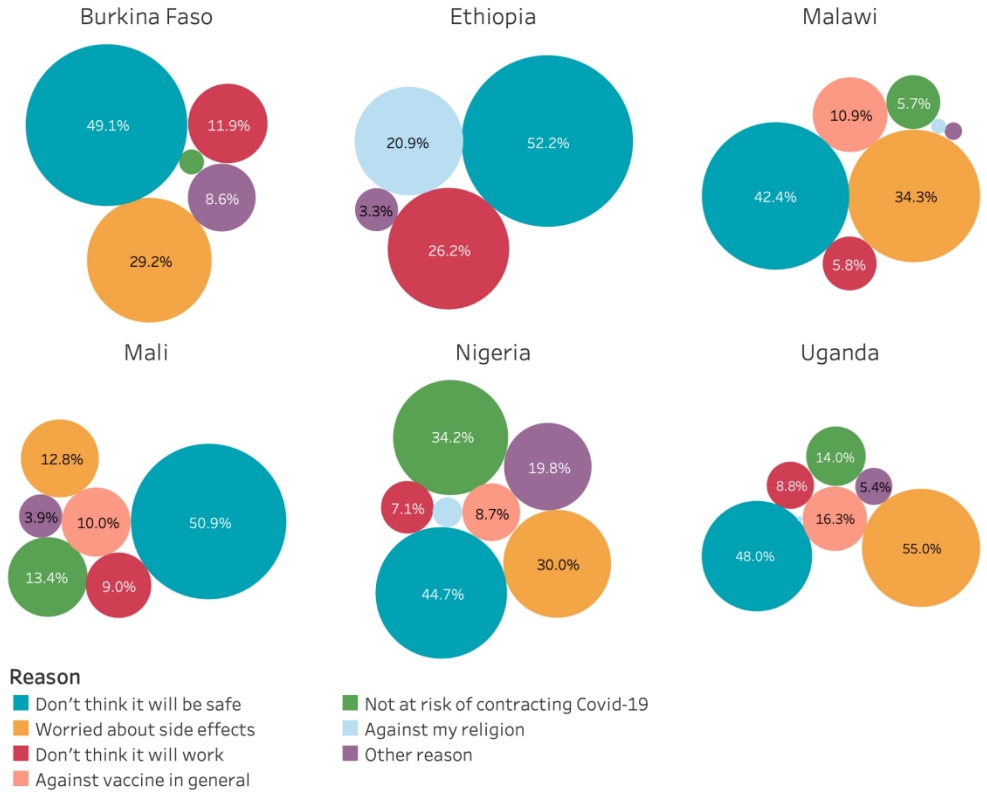

Figure 2: Reasons for covid-19 vaccine hesitancy

321x257mm (144 x 144 DPI)

Appendix

Table A.1. Household selection, number of households

Panel A. Households (N)	**Burkina Faso**	**Ethiopia**	**Malawi**	**Mali**	**Nigeria**	**Uganda**
Pre-COVID-19 FtF survey	7,010	6,770	3,181	7,000	4,976	3,098
With phone numbers	6,877	5,374	2,337	6,300	4,934	2,386
Attempted to contact	2,500	5,374	2,337	2,270	3,000	2,386
Reached	2,062	3,357	1,743	2,270	2,057	2,246
Phone interviews completed	1,945	2,701	1,589	1,765	1,766	2,129

Table A.2.Willingness to get vaccinated by country and pooled, mean and 95% confidence interval

If an approved vaccine to prevent coronavirus was available right now at no cost, would you agree to be vaccinated? —Yes	Mean	Lower Bound	Upper Bound	N
Burkina Faso, weighted	79.6%	77.0%	82.3%	1,945
Ethiopia, weighted	97.9%	97.2%	98.6%	2,701
Malawi, weighted	82.7%	80.0%	85.4%	1,589
Mali, weighted	64.5%	61.3%	67.8%	1,765
Nigeria, weighted	86.2%	83.9%	88.5%	1,766
Uganda, weighted	84.5%	82.2%	86.8%	2,129
Pooled, weighted	87.6%	86.4%	88.8%	11,895
Pooled, unweighted	82.4%	81.7%	83.1%	

For peer review only

For peer review only - http://bmjopen.bmj.com/site/about/guidelines.xhtml

Table A. 3. Unweighted descriptive statistics in the baseline F2F surveys and the HFPS .

	Burkina Faso		Ethiopia		Malawi		Mali		Nigeria		Uganda	
	F2F	HFPS	F2F	HFPS	F2F	HFPS	F2F	HFPS	F2F	HFPS	F2F	HFPS
Men	45.5	78.7	47.3	62.7	47.6	59.8	47.4	78.9	48.3	72.3	48.2	50.7
HH heads	28.7	86.1	38.5	82.5	37.0	74.5	27.1	82.3	32.7	79.3	35.1	74.5
Urban	44.9	72.0	54.0	71.9	26.0	36.8	41.7	66.3	32.0	39.3	24.2	25.8
Primary education	26.1	32.4	34.6	52.9	37.2	50.2	20.2	30.9	66.5	75.1	47.8	51.1
< 30 years	44.1	11.3	49.6	29.4	52.9	27.8	42.6	13.8	42.5	13.5	48.2	15.3
30 - 60 years	45.7	72.2	42.1	62.4	38.2	62.7	45.7	70.4	44.9	69.0	40.0	66.2
60+ years	10.1	16.5	8.3	8.3	8.9	9.5	11.7	15.9	12.6	17.5	11.8	18.6
Quintile 1 (poorest)	18.7	14.0	11.4	4.6	15.7	8.7	18.2	14.6	12.7	9.5	18.2	15.8
Quintile 2	18.9	15.6	13.8	9.5	17.6	14.6	18.9	16.4	14.4	13.1	19.0	18.5
Quintile 3	19.9	17.9	15.0	12.4	17.4	16.1	21.0	19.0	19.2	18.7	20.4	20.7
Quintile 4	21.3	22.5	20.3	22.0	22.2	23.5	20.7	22.2	21.4	21.9	21.7	22.5
Quintile 5 (richest)	21.2	30.0	39.4	51.5	27.1	37.1	21.2	27.8	32.3	36.9	20.7	22.5
**Observations**	24,396	1,742	17,563	2,701	8,588	1,589	24,394	1,765	15,230	1,766	8,763	2,129

Note: The table shows descriptive statistics and sample sizes for the face-to-face (F2F) pre-Covid samples of the general adult population (>15 years) and the high-frequency phone surveys (HFPS) during the pandemic. All values except observation numbers in percent.

For peer review only - http://bmjopen.bmj.com/site/about/guidelines.xhtml

Table A.4. Estimated acceptance rates using household or individual weights.

	**Burkina Faso**		**Ethiopia**		**Malawi**		**Mali**		**Nigeria**		**Uganda**	
	HH	Indiv.	HH	Indiv.	HH	Indiv.	HH	Indiv.	HH	Indiv.	HH	Indiv.
Overall	0.798	0.815	0.979	0.973	0.827	0.828	0.645	0.657	0.862	0.843	0.845	0.844
	(0.0135)	(0.0176)	(0.0036)	(0.0068)	(0.0137)	(0.0176)	(0.0166)	(0.0241)	(0.0118)	(0.0168)	(0.0116)	(0.0129)
Men	0.800	0.839	0.987	0.987	0.846	0.843	0.642	0.666	0.901	0.888	0.854	0.862
	(0.0151)	(0.0178)	(0.0037)	(0.0050)	(0.0162)	(0.0216)	(0.0185)	(0.0281)	(0.0126)	(0.0200)	(0.0160)	(0.0161)
Women	0.788	0.779	0.958	0.949	0.798	0.816	0.657	0.643	0.757	0.793	0.835	0.831
	(0.0304)	(0.0346)	(0.0088)	(0.0164)	(0.0240)	(0.0268)	(0.0374)	(0.0435)	(0.0269)	(0.0282)	(0.0169)	(0.0191)
Urban	0.653	0.695	0.954	0.947	0.700	0.719	0.669	0.668	0.820	0.814	0.810	0.802
	(0.0207)	(0.0298)	(0.0070)	(0.0131)	(0.0318)	(0.0398)	(0.0156)	(0.0324)	(0.0229)	(0.0302)	(0.0232)	(0.0260)
Rural	0.891	0.905	0.991	0.986	0.859	0.859	0.635	0.650	0.881	0.858	0.861	0.862
	(0.0160)	(0.0181)	(0.0040)	(0.0078)	(0.0148)	(0.0193)	(0.0227)	(0.0335)	(0.0135)	(0.0203)	(0.0132)	(0.0147)
Q1	0.914	0.928	0.997	0.997	0.903	0.922	0.640	0.643	0.949	0.957	0.941	0.956
	(0.0237)	(0.0226)	(0.0023)	(0.0020)	(0.0335)	(0.0280)	(0.0382)	(0.0572)	(0.0244)	(0.0250)	(0.0163)	(0.0120)
Q2	0.914	0.923	0.997	0.994	0.923	0.906	0.703	0.690	0.884	0.849	0.905	0.863
	(0.0224)	(0.0309)	(0.0029)	(0.0055)	(0.0183)	(0.0284)	(0.0347)	(0.0582)	(0.0340)	(0.0493)	(0.0202)	(0.0321)
Q3	0.823	0.812	0.971	0.958	0.819	0.775	0.702	0.699	0.911	0.851	0.849	0.856
	(0.0312)	(0.0406)	(0.0136)	(0.0249)	(0.0329)	(0.0549)	(0.0348)	(0.0552)	(0.0196)	(0.0416)	(0.0263)	(0.0263)
Q4	0.800	0.766	0.986	0.984	0.745	0.779	0.602	0.664	0.834	0.840	0.785	0.784
	(0.0304)	(0.0466)	(0.0048)	(0.0052)	(0.0356)	(0.0400)	(0.0392)	(0.0478)	(0.0286)	(0.0334)	(0.0294)	(0.0324)
Q5	0.548	0.606	0.957	0.949	0.797	0.819	0.582	0.594	0.809	0.798	0.799	0.796
	(0.0312)	(0.0477)	(0.0082)	(0.0171)	(0.0256)	(0.0282)	(0.0361)	(0.0484)	(0.0218)	(0.0304)	(0.0249)	(0.0282)

Note: The table compares estimated acceptance rates for a free, approved covid-19 vaccine for different demographics. It distinguishes between using weights that correct for coverage and non-response bias at the household-level (household weights) and, where available, using individual-level phone weights that also correct for respondent selection at the individual-level (individual weights). The samples do not comprise observations for which no individual weight could be calibrated due to missing baseline information. Standard errors in parentheses.

Table A.5. Logistic regression on the correlates of hesitancy using individual-level survey weights, marginal effects.

VARIABLES	(1) Burkina Faso	(2) Ethiopia	(3) Malawi	(4) Mali	(5) Nigeria	(6) Uganda
Respondent is male	0.0265	0.0125	0.0272	0.116**	0.0307	0.00464
	(0.0351)	(0.00942)	(0.0371)	(0.0494)	(0.0406)	(0.0244)
Lives in urban area	-0.0750**	-0.0236**	-0.0930***	-0.0221	-0.00481	0.00853
	(0.0323)	(0.0116)	(0.0348)	(0.0388)	(0.0370)	(0.0223)
Years of education	-0.00462	-0.00154	-0.0123***	-0.00337	-0.00657	-0.00261
	(0.00293)	(0.000938)	(0.00417)	(0.00356)	(0.00410)	(0.00247)
Age group, 30-60 yrs	0.0354	-0.00114	-0.00707	-0.0117	-0.0124	-0.0340
	(0.0490)	(0.00984)	(0.0387)	(0.0476)	(0.0356)	(0.0294)
Age group, 60+ yrs	0.124**	0.00134	-0.0331	-0.0471	-0.0321	0.0134
	(0.0491)	(0.0196)	(0.0720)	(0.0612)	(0.0628)	(0.0317)
Household head	-0.00843	0.0226*	-0.00400	-0.0972*	0.0333	0.0204
	(0.0401)	(0.0119)	(0.0385)	(0.0549)	(0.0415)	(0.0267)
Household Size	0.00287	0.00186	0.00971	-0.00534*	0.00834	0.00268
	(0.00510)	(0.00181)	(0.00842)	(0.00322)	(0.00591)	(0.00564)
Expenditure, 2nd quintile	0.00107	0.00591	-0.0203	0.0270	-0.0994	-0.0871***
	(0.0502)	(0.0131)	(0.0469)	(0.0638)	(0.0757)	(0.0323)
Expenditure, 3rd quintile	-0.0660	-0.0277	-0.178**	0.0325	-0.0371	-0.0813***
	(0.0428)	(0.0193)	(0.0712)	(0.0537)	(0.0666)	(0.0287)
Expenditure, 4th quintile	-0.0921**	-0.00480	-0.118**	-0.0504	-0.0428	-0.122***
	(0.0466)	(0.0118)	(0.0522)	(0.0595)	(0.0588)	(0.0316)
Expenditure, 5th (richest) quintile	-0.123**	-0.0157	-0.0143	-0.115*	-0.0679	-0.103***
	(0.0528)	(0.0126)	(0.0461)	(0.0684)	(0.0631)	(0.0317)
Willing to be tested for covid-19	0.271***	0.0839***	0.249***	0.468***	0.224***	0.348***
	(0.0287)	(0.0149)	(0.0440)	(0.0171)	(0.0302)	(0.0287)
HH received assistance during covid-19	0.0840*	0.0177	-0.0412	0.0406	-0.00112	0.0597***
	(0.0505)	(0.0127)	(0.0455)	(0.0598)	(0.0328)	(0.0214)
Gov't not trustworthy			-0.0408			
			(0.0322)			
Satisfied with gov't response				0.0356	0.0235	
				(0.0446)	(0.0325)	
Observations	1,738	2,654	1,542	1,590	1,700	2,106
Pseudo R2	0.225	0.357	0.136	0.325	0.162	0.238

Robust standard errors in parentheses. *** p<0.01, ** p<0.05, * p<0.1. Weighted logit regressions with willingness to take a free, approved covid-19 vaccine as dependent variable.

For peer review only - http://bmjopen.bmj.com/site/about/guidelines.xhtml

Table A.6: Bivariate logistic regression on government trust and satisfaction using household-level survey weights.

VARIABLES	(1) Malawi	(2) Mali	(3) Nigeria
Government not trustworthy	-0.0638**		
	(0.0322)		
Satisfied with government response		0.161***	0.0568**
		(0.0552)	(0.0232)
Observations	1,589	1,631	1,766
Controls	YES	YES	YES
Pseudo R2	0.00574	0.00742	0.00847

Robust standard errors in parentheses. *** p<0.01, ** p<0.05, * p<0.1

For peer review only

Reporting checklist for cross sectional study.

Based on the STROBE cross sectional guidelines.

von Elm E, Altman DG, Egger M, Pocock SJ, Gotzsche PC, Vandenbroucke JP. The Strengthening the Reporting of Observational Studies in Epidemiology (STROBE) Statement: guidelines for reporting observational studies.

		Reporting Item	Page Number
**Title and abstract**			
Title	#1a	Indicate the study's design with a commonly used term in the title or the abstract	1
Abstract	#1b	Provide in the abstract an informative and balanced summary of what was done and what was found	1,2
**Introduction**			
Background / rationale	#2	Explain the scientific background and rationale for the investigation being reported	3
Objectives	#3	State specific objectives, including any prespecified hypotheses	1,3
**Methods**			
Study design	#4	Present key elements of study design early in the paper	3-5

For peer review only

Setting	#5	Describe the setting, locations, and relevant dates, including periods of recruitment, exposure, follow-up, and data collection	4
Eligibility criteria	#6a	Give the eligibility criteria, and the sources and methods of selection of participants.	4-5
	#7	Clearly define all outcomes, exposures, predictors, potential confounders, and effect modifiers. Give diagnostic criteria, if applicable	4
Data sources / measurement	#8	For each variable of interest give sources of data and details of methods of assessment (measurement). Describe comparability of assessment methods if there is more than one group. Give information separately for for exposed and unexposed groups if applicable.	4-5
Bias	#9	Describe any efforts to address potential sources of bias	5
Study size	#10	Explain how the study size was arrived at	4-5
Quantitative variables	#11	Explain how quantitative variables were handled in the analyses. If applicable, describe which groupings were chosen, and why	5
Statistical methods	#12a	Describe all statistical methods, including those used to control for confounding	5
Statistical methods	#12b	Describe any methods used to examine subgroups and interactions	5

For peer review only

Statistical methods	#12c	Explain how missing data were addressed	18
Statistical methods	#12d	If applicable, describe analytical methods taking account of sampling strategy	5
Statistical methods	#12e	Describe any sensitivity analyses	5
**Results**			
Participants	#13a	Report numbers of individuals at each stage of study—eg numbers potentially eligible, examined for eligibility, confirmed eligible, included in the study, completing follow-up, and analysed. Give information separately for for exposed and unexposed groups if applicable.	16
Participants	#13b	Give reasons for non-participation at each stage	16
Participants	#13c	Consider use of a flow diagram	n/a
Descriptive data	#14a	Give characteristics of study participants (eg demographic, clinical, social) and information on exposures and potential confounders. Give information separately for exposed and unexposed groups if applicable.	17
Descriptive data	#14b	Indicate number of participants with missing data for each variable of interest	n/a

For peer review only

Outcome data	#15	Report numbers of outcome events or summary measures. Give information separately for exposed and unexposed groups if applicable.	16
Main results	#16a	Give unadjusted estimates and, if applicable, confounder-adjusted estimates and their precision (eg, 95% confidence interval). Make clear which confounders were adjusted for and why they were included	6,16
Main results	#16b	Report category boundaries when continuous variables were categorized	8
Main results	#16c	If relevant, consider translating estimates of relative risk into absolute risk for a meaningful time period	n/a
Other analyses	#17	Report other analyses done—e.g., analyses of subgroups and interactions, and sensitivity analyses	7-8,10-11, 18-20
**Discussion**			
Key results	#18	Summarise key results with reference to study objectives	9
Limitations	#19	Discuss limitations of the study, taking into account sources of potential bias or imprecision. Discuss both direction and magnitude of any potential bias.	10-11
Interpretation	#20	Give a cautious overall interpretation considering objectives, limitations, multiplicity of analyses, results from similar studies, and other relevant evidence.	11

For peer review only

Generalisability	#21	Discuss the generalisability (external validity) of the study results	10-11
**Other Information**			
Funding	#22	Give the source of funding and the role of the funders for the present study and, if applicable, for the original study on which the present article is based	12

Notes:

- 17: 7-8,10-11, 18-20 The STROBE checklist is distributed under the terms of the Creative Commons Attribution License CC-BY. This checklist was completed on 22. June 2021 using https://www.goodreports.org/, a tool made by the EQUATOR Network in collaboration with Penelope.ai

For peer review only

BMJ Open

The Acceptance of COVID-19 Vaccines in Sub-Saharan Africa: Evidence from Six National Phone Surveys

Journal:	*BMJ Open*
Manuscript ID	bmjopen-2021-055159.R1
Article Type:	Original research
Date Submitted by the Author:	19-Aug-2021
Complete List of Authors:	Kanyanda, Shelton; World Bank Group, Development Data Group<br>Markhof, Yannick; World Bank Group, Development Data Group<br>Wollburg, Philip; World Bank Group, Development Data Group<br>Zezza, Alberto; World Bank Group, Development Data Group
**Primary Subject Heading**:	Public health
Secondary Subject Heading:	Health policy
Keywords:	COVID-19, PUBLIC HEALTH, Health policy < HEALTH SERVICES ADMINISTRATION & MANAGEMENT

SCHOLARONE™ Manuscripts

I, the Submitting Author has the right to grant and does grant on behalf of all authors of the Work (as defined in the below author licence), an exclusive licence and/or a non-exclusive licence for contributions from authors who are: i) UK Crown employees; ii) where BMJ has agreed a CC-BY licence shall apply, and/or iii) in accordance with the terms applicable for US Federal Government officers or employees acting as part of their official duties; on a worldwide, perpetual, irrevocable, royalty-free basis to BMJ Publishing Group Ltd ("BMJ") its licensees and where the relevant Journal is co-owned by BMJ to the co-owners of the Journal, to publish the Work in this journal and any other BMJ products and to exploit all rights, as set out in our licence.

The Submitting Author accepts and understands that any supply made under these terms is made by BMJ to the Submitting Author unless you are acting as an employee on behalf of your employer or a postgraduate student of an affiliated institution which is paying any applicable article publishing charge ("APC") for Open Access articles. Where the Submitting Author wishes to make the Work available on an Open Access basis (and intends to pay the relevant APC), the terms of reuse of such Open Access shall be governed by a Creative Commons licence – details of these licences and which Creative Commons licence will apply to this Work are set out in our licence referred to above.

Other than as permitted in any relevant BMJ Author's Self Archiving Policies, I confirm this Work has not been accepted for publication elsewhere, is not being considered for publication elsewhere and does not duplicate material already published. I confirm all authors consent to publication of this Work and authorise the granting of this licence.

For peer review only

The Acceptance of COVID-19 Vaccines in Sub-Saharan Africa: Evidence from Six National Phone Surveys

Shelton Kanyanda[1], Yannick Markhof[2], Philip Wollburg[3], Alberto Zezza[4]*

[1] Consultant, World Bank, Development Data Group

[2] Consultant, World Bank, Development Data Group

[3] Economist, World Bank, Development Data Group

[4] Program Manager, World Bank, Development Data Group

* corresponding author

azezza@worldbank.org

For peer review only

Keywords: vaccines, COVID-19, Africa, vaccine hesitancy, vaccine acceptance

Word count: 4,124

Abstract

Objectives

To estimate the willingness to accept a COVID-19 vaccine in six Sub-Saharan African countries and identify differences in acceptance across countries and population groups.

Design

Cross-country comparable, descriptive study based on a longitudinal survey.

Setting

Six national surveys from countries representing 38% of the Sub-Saharan African population (Burkina Faso, Ethiopia, Malawi, Mali, Nigeria, and Uganda).

Participants

Respondents of national high-frequency phone surveys (HFPS), aged 15 and older, drawn from a nationally representative sample of households.

Main outcome measures

Willingness to get vaccinated against COVID-19 if an approved vaccine is provided now and for free, disaggregated by demographic attributes and socio-economic factors obtained from national household surveys. Correlates of and reasons for vaccine hesitancy.

Results

Acceptance rates in the six Sub-Saharan African countries studied are generally high, with at least four in five people willing to be vaccinated in all but one country. Vaccine acceptance ranges from nearly universal in Ethiopia (97.9%, 97.2% to 98.6%) to below what would likely be required for herd immunity in Mali (64.5%, 61.3% to 67.8%). We find little evidence for systematic differences in vaccine hesitancy by sex or age but some clusters of hesitancy in urban areas, among the better educated, and in richer households. Safety concerns about the vaccine in general and its side effects specifically emerge as the primary reservations toward a COVID-19 vaccine across countries.

Conclusions

Our findings suggest that inadequate demand is unlikely to represent the key bottleneck to reaching high COVID-19 vaccine coverage in Sub-Saharan Africa. To turn intent into effective demand, targeted information, sensitization, and engagement campaigns bolstering confidence in the safety of approved vaccines and reducing concerns about side effects will be crucial to safeguard the swift progression of vaccine rollout in one of the world's poorest regions.

Article Summary

Strengths and limitations of this study

- Much of the current debate on vaccination campaigns in low-income countries in general and Sub Saharan Africa (SSA) in particular focuses on supply-chain and financial factors, yet there is a dearth of robust, comparable evidence on COVID-19 vaccine hesitancy in these countries.
- We use cross-country comparable data from six national high-frequency phone surveys in SSA to fill this crucial knowledge gap
- Our sample is drawn from large, nationally representative sampling frames that provide a rich set of demographic and socio-economic characteristics by which we disaggregate our analysis
- Through a set of re-calibrated survey weights, our analysis adjusts for the selection biases common in other remote surveys, yet, some bias may persist
- Attitudes toward vaccines are malleable and may change over time

Introduction

As vaccination campaigns in high-income countries are accelerating, large swathes of the global population living in low- and middle-income countries remain severely exposed to the novel coronavirus disease (COVID-19).[1] Sub-Saharan Africa (SSA) is home to 433 million people living below the international absolute poverty line (about two thirds of the global poor population).[2] For these populations, non-pharmaceutical interventions to curb the spread of the disease impinged on already precarious livelihoods.[3] Furthermore, the region is characterized by high prevalence of comorbidities, and public health systems that are ill equipped to stave off the burden of mass infection.[1,3,4] Reaching large-scale vaccination coverage in SSA is thus pivotal in the global effort to halt the spread of the disease and limit its toll on lives and livelihoods.[5–7]

Recent calls to action by international stakeholders and a growing body of scholarly research focus on supply-chain and financial factors to safeguard sufficient vaccine availability in SSA. The COVAX initiative aims to provide doses sufficient to vaccinate up to 20% of the population in the region, far below the target population required for a coverage of 60%, or 321.5 million individuals.[8,9] Still, this would leave an estimated financing gap of over $10 billion in SSA.[10] A June 2021 resolution from the G7 pledging one billion COVID-19-vaccine doses for low-income countries offers hope for those supply gaps being filled. While these considerations concern the availability of vaccines, another key factor for the success of vaccination campaigns in SSA is the willingness to be vaccinated within the population. Yet, there is a dearth of large-scale evidence on vaccine acceptance in low-income countries in general and SSA in particular where previous studies came to diverging conclusions.[11–14]

Based on a cross-country comparable sample with national scope from six Sub-Saharan African countries, we fill this knowledge gap by providing estimates of vaccine acceptance for a population representative of around 416 million people, 38% of the population of Sub-Saharan Africa.[15] Drawing on the high-frequency phone surveys (HFPS) based on pre-pandemic sampling frames from nationally representative, face-to-face household surveys supported by the World Bank's Living Standards Measurement Study – Integrated Survey on Agriculture (LSMS-ISA), we are able to link COVID-19 vaccine acceptance rates to a rich set of demographic and socio-economic characteristics. Along with recalibrated sampling weights, this allows our study to provide robust insights into the likelihood of the current efforts to ensure sufficient supply of vaccination doses to also meet adequate demand in SSA, identify clusters of hesitancy, and contribute to a swift rollout of vaccination campaigns where they will be needed most.

Methods

Data and survey instrument

We use data from the national longitudinal high-frequency phone surveys on COVID-19 (HFPS) conducted in Burkina Faso, Ethiopia, Malawi, Mali, Nigeria, and Uganda. Survey implementation was led by the respective national statistical agencies, the Burkina Faso National Institute of Statistics and Demography; Laterite Ethiopia in collaboration with the Central Statistical Agency of Ethiopia; Malawi National Statistical Office; Mali National Institute of Statistics; Nigeria

For peer review only

Bureau of Statistics; and Uganda Bureau of Statistics; and supported by the World Bank Living Standards Measurement Study and the Poverty and Equity Global Practice. The HFPS on COVID-19 have been implemented monthly since May 2020, aiming to gauge the impact of the COVID-19 pandemic on individual and household attitudes, socioeconomic and health outcomes.[16] Section 2 of the supplementary appendix includes more detail on the conception and implementation of the HFPS.

The HFPS rounds in which vaccine hesitancy was covered in a cross-country comparable fashion were conducted in September 2020 (Ethiopia, round 6), October-November 2020 (Malawi, round 5; Mali, round 5; and Nigeria, round 6) and December 2020 (Burkina Faso, round 5; and Uganda, round 4). The primary survey questions of interest, posed to each phone survey respondent, was: '*If an approved vaccine to prevent coronavirus was available right now at no cost, would you agree to be vaccinated?'* with response options '*yes*', '*no*', and '*not sure'*. If respondents answered '*no*' or *'not sure'*, this was followed up by a question about possible reasons for refusing to be vaccinated: '*What are the reasons you would not agree to be vaccinated?'* with response options:

(1) 'I don't think it will be safe'
(2) 'I am worried about the side effects'
(3) 'It is against my religion'
(4) 'I am not at risk of contracting COVID-19'
(5) 'I don't think it will work'
(6) 'I am against vaccine in general'
(7) Other

We are interested in the association between willingness to be vaccinated and a standard set of demographic and socioeconomic variables, such as gender, age, income, and education. These are available for respondents either from the HFPS directly or from pre-COVID-19 face-to-face (FtF) surveys, which served as the sampling frames for the HFPS. We further draw on multiple rounds of the HFPS to create variables on respondents' attitudes towards the COVID-19 emergency and how it has affected them and their families. These include willingness to be tested for COVID-19, rating of government response, and whether households received any government assistance during the pandemic. The data as well as survey instruments and basic information documents are available publicly on the World Bank Microdata Library under the High-Frequency Phone Survey collection.[17]

Sampling and sample representativeness

The samples for the HFPS were drawn from mobile phone numbers recorded during data collection for nationally representative face-to-face (FtF) household surveys implemented prior to the COVID-19 pandemic with support from the World Bank Living Standards Measurement Study – Integrated Surveys on Agriculture (LSMS-ISA) program. Specifically, the Burkina Faso Enquête harmonisée sur les conditions de vie des ménages 2018/19, Ethiopia Socioeconomic Survey 2018/19, Malawi Integrated Household Panel Survey 2019, Mali Enquête harmonisée sur les conditions de vie des ménages 2018/19, Nigeria General Household Survey – Panel 2018/19, and Uganda National Panel Survey 2019/20. At least one phone number was recorded for all households with access to a phone, including through a contact person outside the household, such

For peer review only

as a friend or neighbor. For the HFPS, Ethiopia, Malawi, and Uganda attempted to contact all households with available phone numbers, while Burkina Faso, Nigeria and Mali selected a random sub-sample of phone numbers to call (Table A.1).

In the absence of universal access to a mobile phone, the HFPS households are likely to be selected samples of the nationally representative FtF survey samples of households.[18] The publicly available HFPS data sets therefore come with recalibrated household survey weights to counteract potential selection biases. The recalibration model takes advantage of the rich information on households with and without access to a phone from the pre-COVID-19 FtF surveys [3,19] and follows a methodology proposed in a reference methodological paper on this subject.[20] The covariates included in the logistic response propensity model included household characteristics such as household size, urban/rural residence, economic status, education, sex and age of the household head, and a number of other household characteristics selected based on their observed bias in the interviewed sample. The effectiveness of this weight recalibration in overcoming selection biases has been documented in HFPS data from four of the six countries we study, such that household-level estimates based on the HFPS data can be broadly considered representative at the national level.[18]

Further, the HFPS survey questions on vaccinations were asked only to the household's main respondent, who had to be 15 or older, as it is impractical to interview all individuals in a household in a phone survey. The selection of main respondents was not randomized so that the group of respondents is likely not representative of the general population of adults at the individual level. Rather, respondents tend to be household heads or their spouses, better educated and slightly older, as a recent study documents.[21] An additional recalibration of survey weights for individual-level analysis analogous to the approach described above improves representativeness, but cannot overcome respondent selection biases in all variables and increases the variance of the estimates.[21]

Considering these potential shortfalls in the representativeness of our data, we present our main results first with the standard HFPS household weights and then, as a robustness and sensitivity check for potential respondent selection biases, with the recalibrated individual-level weights. A summary of unweighted individual characteristics of FtF adults vis-à-vis HFPS adults is presented in Table A.2.

Statistical analysis

The statistical analysis proceeds in several steps. First, we estimate the weighted mean of willingness to get vaccinated by country, and within countries by sex of respondent, residence (urban and rural) and income quintile, as well as reasons for vaccine hesitancy, using the recalibrated household weights. Second, we explore how individual and household characteristics, such as education and expenditure, correlate with the willingness to get vaccinated in a set of multivariate logit regressions, again using household weights. To assess how much the differences in the attributes of respondents vis-à-vis the general adult population may affect the representativeness of our results, we assess the sensitivity of the results to using individual-level recalibrated weights instead of the public-use household weights (see Discussion).[21]

For peer review only

Patient and public involvement

This research did not involve consultation with patients or the public.

Results

Descriptive results

Overall, we find high levels of willingness to be vaccinated: Acceptance is estimated to be nearly universal in Ethiopia at 97.9% (95% confidence interval 97.2% to 98.6%) and very high in Nigeria (86.2%, 83.9% to 88.5%), Uganda (84.5%, 82.2% to 86.8%), Malawi (82.7%, 80.0% to 85.4%), and Burkina Faso (79.5%, 76.9% to 82.1%). In these countries at least four in five respondents would agree to be vaccinated if an approved vaccine was made available for free to them (Figure 1, Panel A). Acceptance is somewhat lower only in Mali where less than two-thirds of respondents (64.5%, 61.3% to 67.8%) reported their willingness to be vaccinated. Pairwise tests of statistical significance of the difference in hesitancy between countries are reported in Table A.3. Notably, Mali is also the only country in which a non-negligible share (12.4%) is uncertain about their answer, a fact that accounts for the majority of the lower acceptance rates in Mali. For the following analysis, we focus on acceptance rates and do not distinguish between respondents rejecting to be vaccinated and those that are not sure. Pooling the data from all countries and weighting them by their respective popualtion sizes yields an overall mean acceptance rate of 87.6% (86.4% to 88.8%) across the six countries (Table A.4).

In Panel B of Figure 1, we disaggregate acceptance rates by respondent sex. Except for Nigeria where acceptance is statistically significantly higher among male than female respondents (90.1%, 87.6% to 92.6% and 75.7%, 70.4% to 80.9%, respectively) and in Ethiopia where there is a small, yet statistically significant difference (98.7%, 97.9% to 99.4% and 95.8%, 94.1% to 97.5%, respectively), we do not find answers to differ between men and women. Similarly, Panel C reports differences in willingness to be vaccinated against COVID-19 between rural and urban areas with higher acceptance in rural areas in Burkina Faso, Ethiopia, and Malawi at the 95% confidence level. One distinct feature of our data is the ability to tap into the rich pre-COVID-19 baseline data from the face-to-face household surveys that served as sampling frames. This way, we are able to determine respondent household's position in the national expenditure distribution and disaggregated acceptance rates by expenditure quintile (Panel D). We generally find a downward sloping pattern in which acceptance is higher among poorer households and lowest among the richest households. This pattern is particularly evident in Burkina Faso but also noteworthy in Uganda and Nigeria. Conversely, acceptance is high throughout all expenditure quintiles in Ethiopia and not significantly lower for the richest two quintiles in Mali.

For those respondents who were hesitant to be vaccinated, the survey asked to provide a reason. From Figure 2, it is evident that safety concerns were paramount ranging from 53.7% (36.1% to 71.3%) of those reporting they would refuse to be vaccinated in Burkina Faso to 42.4% (33.8% to 50.9%) in Malawi, despite the wording of the questions making it explicit that the vaccine would be officially approved. Other notable reasons include worries about the potential side effects of the

For peer review only

vaccine (between 55% (46.9% to 63.0%) in Uganda and 12.8% (9.1% to 16.5%) in Mali) and the belief not to be at risk of contracting the virus (34.2% (25.2% to 43.3%) in Nigeria to 0.1% (0% to 2.1%) in Burkina Faso).

Correlates of hesitancy

Exploiting the richness of our data, we can explore correlational patterns between willingness to be vaccinated for COVID-19 and a large set of respondent and household characteristics. Table 1 confirms in a multivariate setting the patterns observed graphically earlier. In general, men tend to be more willing to take the vaccine, though we only detect a statistically significant effect in Nigeria. Respondents in urban areas are more skeptical with significant differences in Burkina Faso, Ethiopia, and Malawi.

An association we observe across countries is between education and vaccine hesitancy. In Burkina Faso, Ethiopia, Malawi, and Nigeria, those with more years of education are significantly less willing to be vaccinated while coefficient signs point in the same direction in Mali and Uganda but are not significant. Lastly, we do not find a pattern according to respondents' age and a mixed picture according to the role of the respondent in the household. Here, household heads which constitute the majority of our sample (see Table A.2) are more willing to be vaccinated in Ethiopia, more hesitant in Mali, and not significantly different from other household members in the remaining four cases.

A pattern we observe in several countries is a higher hesitancy toward the vaccine in richer households compared to the poorest expenditure quintile, with coefficients significant in Mali and Uganda for the richest two quintiles, for the top quintile in Burkina Faso and Nigeria, and the third and fourth quintiles in Malawi.

The data further show a strong association between vaccine hesitancy and the willingness to be tested for COVID-19 suggesting similar underlying reasons for (or against) testing and getting vaccinated. Another hypothesis we explore is whether there is an association between the receipt of some assistance during the pandemic, e.g. through one of the large-scale social protection programs launched to combat the fallout of the pandemic, and willingness to receive a free, approved vaccine against COVID-19. While coefficient signs mostly point to a positive association, the effect is not statistically significantly different from zero despite all countries in our sample launching some social protection response to the crisis.[22]

A last hypothesis we pursue concerns the relationship between trust in and satisfaction with the government and willingness to be vaccinated. For example, more skeptical individuals toward the government or those dissatisfied with its crisis management may be more reluctant to accept a state-provided vaccine.[12,23] We only have data on trust in the government's crisis management in Malawi and satisfaction in Mali and Nigeria but find our hypotheses are confirmed in bivariate logistic regressions (Table A.5). However, after controlling for all other factors in Table 1, the coefficients are no longer significant.

For peer review only

Table 1: Correlates of hesitancy.

Correlates of hesitancy - marginal effects from multivariate logit regressions

VARIABLES	(1) Burkina Faso	(2) Ethiopia	(3) Malawi	(4) Mali	(5) Nigeria	(6) Uganda
Respondent is male	-0.0388	0.00913	0.0419	0.0522	0.0932***	0.0179
	(0.0340)	(0.00569)	(0.0311)	(0.0413)	(0.0268)	(0.0224)
Lives in urban area	-0.0878***	-0.0191**	-0.0880***	0.0237	-0.0151	0.0168
	(0.0294)	(0.00858)	(0.0289)	(0.0286)	(0.0222)	(0.0212)
Years of education	-0.00610**	-0.00137*	-0.0135***	-0.00452	-0.00680**	-0.00164
	(0.00252)	(0.000734)	(0.00330)	(0.00288)	(0.00286)	(0.00283)
Age group 30-60 yrs	-0.0166	0.00494	0.0152	0.0297	0.0436	-0.0274
	(0.0365)	(0.00838)	(0.0312)	(0.0458)	(0.0366)	(0.0269)
Age group 60+ yrs	0.0446	0.00700	-0.0140	-0.0273	0.0499	0.0179
	(0.0435)	(0.0141)	(0.0505)	(0.0584)	(0.0461)	(0.0341)
Household head	0.0558	0.0171**	-0.00894	-0.0886**	-0.0266	-0.00563
	(0.0376)	(0.00776)	(0.0381)	(0.0450)	(0.0325)	(0.0242)
Household Size	0.00310	0.000705	0.0103*	-0.00536	0.00286	0.00631
	(0.00378)	(0.00152)	(0.00557)	(0.00358)	(0.00580)	(0.00476)
Expenditure, 2nd quintile	0.0230	0.00677	0.0198	0.0307	-0.0677	-0.0302
	(0.0426)	(0.0102)	(0.0437)	(0.0441)	(0.0564)	(0.0319)
Expenditure, 3rd quintile	-0.0568	-0.0216	-0.0953*	0.0166	0.00195	-0.0500
	(0.0430)	(0.0195)	(0.0516)	(0.0431)	(0.0427)	(0.0308)
Expenditure, 4th quintile	-0.0357	-0.00381	-0.116**	-0.0932*	-0.0623	-0.107***
	(0.0442)	(0.0103)	(0.0473)	(0.0501)	(0.0458)	(0.0340)
Expenditure, 5th (richest) quintile	-0.164***	-0.0111	-0.0106	-0.135**	-0.0696*	-0.0754**
	(0.0534)	(0.0108)	(0.0457)	(0.0536)	(0.0421)	(0.0346)
Willing to be tested for COVID-19	0.208***	0.0619***	0.253***	0.461***	0.221***	0.340***
	(0.0313)	(0.00889)	(0.0389)	(0.0172)	(0.0221)	(0.0254)
HH received assistance during COVID-19	0.0490	0.00607	-0.00457	-0.00728	0.0152	0.0307
	(0.0470)	(0.00886)	(0.0322)	(0.0618)	(0.0220)	(0.0194)
Gov't not trustworthy			-0.0455			
			(0.0291)			
Satisfied with gov't response				0.0354	0.0279	
				(0.0450)	(0.0202)	
Observations	1,742	2,654	1,542	1,591	1,703	2,106
Pseudo R2	0.183	0.308	0.137	0.265	0.218	0.233

Robust standard errors in parentheses. *** p<0.01, ** p<0.05, * p<0.1. Weighted logit regressions with willingness to take a free, approved vaccine as dependent variable.

For peer review only

Discussion

Principal findings

Our study uses cross-country comparable data from the national longitudinal high frequency phone surveys on COVID-19 (HFPS) in six Sub-Saharan African countries to estimate acceptance rates for an approved, free COVID-19 vaccine. By linking phone survey data to the nationally representative, large-scale, face-to-face household surveys that served as sampling frames for the HFPS, we provide robust estimates of the willingness to be vaccinated against COVID-19 across a diverse set of demographics and respondent and household characteristics. Our headline results indicate high acceptance rates with at least four in five respondents signaling their willingness to be vaccinated in all but one of the countries studied. There is cross-country variation, with willingness to be vaccinated ranging from 64.5% in Mali (61.3% to 67.8%), where a further 12.4% are yet undecided, to near universal acceptance in Ethiopia (97.9%, 97.2% to 98.6%). We also find little evidence for systematic differences in vaccine hesitancy across gender or age but some notable clusters of hesitancy in urban areas, among those better educated, and in richer households. Across countries, safety concerns about the vaccine in general and its side effects specifically emerged as the primary reservations toward a COVID-19 vaccine.

Strengths and comparison with other studies

In relation to the existing literature, our study has several advantages in the domains of coverage and sample selection, richness of the data, and analytical depth. In terms of coverage, our study focuses on a region with yet scant evidence on willingness to be vaccinated against COVID-19. Importantly, the data we assemble are cross-country comparable and without exception based on large, nationally representative sampling frames from pre-COVID, face-to-face household surveys. This allows us to calibrate survey weights that adjust for the coverage and non-response biases that plague other studies at a time when regular face-to-face data collection nearly came to a complete halt.[24–26] Furthermore, our study is unique in that the pre-existing survey data from which our samples are drawn allow us to tap into a rich set of baseline individual and household characteristics. This facilitates a more rigorous analysis and disaggregation of acceptance rates, including, for instance, households' position in the national (pre-crisis) expenditure distribution, than has been possible previously. Our study thus stands out as a fully cross-country comparable, multivariate, and inferential analysis of vaccine hesitancy on the African continent.

Compared to our study, previous studies that analyze COVID-19 vaccine acceptance rates and the reasons for refusal predominantly (i) focus on middle- and high-income countries, (ii) represent single-country or not cross-country comparable samples, (iii) study particular subpopulations such as university students, healthcare workers, or participants of unrelated pre-COVID studies, (iv) rely on non-random sampling, or (v) can only draw on a small set of demographics and characteristics to disaggregate their analysis.[14,21,27–37]

A cross-country study of 19 countries with samples obtained from commercial online panel providers found 71.5% of respondents willing to be vaccinated against COVID-19 with a rate of 65.2% in Nigeria as the only low-income country.[38] Similarly, a literature review of 31 studies

For peer review only - http://bmjopen.bmj.com/site/about/guidelines.xhtml

covering 33 countries found COVID-19 vaccine acceptance rates at or above 70% among studies focusing on the general population but also large regional and intra-regional differences and a dearth of evidence particularly from Sub-Saharan Africa.[39]

Few studies explicitly focus on acceptance rates in the poorest countries. Assembling an amalgamation of data samples with different sources, sampling methodologies, and coverage, one study finds generally high acceptance rates in ten LMICs in Asia, Africa, and South America.[13] Among countries also covered in our study, acceptance rates are lower than what we find in Burkina Faso (66.5%, national phone sample obtained by random digit dialing) and Nigeria (76.2%, random sample of residents of one state from telephone list), close in urban Uganda (76.5%, random sample of households in Kampala), and very similar in rural Uganda (85.8%, non-random sample of women in 13 districts).[13] A second study in nine LMICs (including DR Congo, Benin, Malawi, Mali and Uganda from SSA) draws on a convenience sample recruited through social media and other online channels.[14] The acceptance rates found heavily depend on the hypothesized vaccine effectiveness but are generally low (below 75%) in all countries from SSA except Uganda. Sample sizes are roughly 5% of ours. Furthermore, the ability to correct for various sample selection biases is likely limited in these studies in the absence of baseline nationally representative sampling frames. Another study in 15 African countries, including Burkina Faso, Malawi, Nigeria, Uganda (all face-to-face interviewing), and Ethiopia (telephone survey) found estimates for the countries also covered in our study mostly in the vicinity of our weighted figures (Ethiopia 94%, Uganda 87.5%, Burkina Faso 86%, Malawi 82.7%, Nigeria 76%).[11,40] Notably, average sample sizes are roughly half of our study's and there is a lack of analytical detail impeding a robust inference of cross-country and -demographic findings. Lastly, our results contrast with a recent Afrobarometer study based on in-person interviews in five West African countries, none of which are also included in our sample. In relation to our study, the study's analysis is purely descriptive and reports low acceptance rates of 40% on average.[12] Similar to what we find, the study cites trust in vaccine safety as the key driver of hesitancy, a result that is further corroborated in the literature.[12–14,30,38,40] Furthermore, the small systematic differences we observe according to gender and age are in line with most previous findings in low-income countries as is a tendency for higher acceptance in rural areas.[12,13,38,40] As with overall acceptance rates, our finding of higher hesitancy among the more educated is in line with two of the studies covering Sub-Saharan Africa [11,13] while another study finds mixed evidence.[12] No other study we are aware of across LMICs (and in fact few across countries of any income classification) assesses vaccine acceptance according to economic status in a manner comparable with the expenditure data we can access from the pre-COVID-19 sampling frames.

Limitations of this study

This study uses data from high-frequency phone surveys (HFPS) with national coverage along with sampling weights specifically recalibrated for the phone surveys to be nationally representative. The weights were shown to be effective at achieving nationally representative estimates at the household level.[18] However, willingness to be vaccinated is primarily not a household-level attribute but an individual decision, though it is reasonable to expect considerable intra-household correlation in attitudes towards vaccination. Phone survey respondents in our data

For peer review only

are not specifically selected to be representative of all individuals and that may limit the population-level representativeness of the results we report. Recalibration of survey weights at the individual level can partially but not fully address this concern.[21] To gauge how sensitive our results are to respondent selection biases in individual-level data, we compare the estimates with household-level weights to the same estimates with individual-level weights. In this test, large deviations between those two sets of estimates would indicate that selection biases affect our results in a fundamental way. However, we find only limited change in the estimates, regardless of which weight is used: in over three-quarters of the cases, deviations do not exceed two percentage points, including the headline findings on willingness to be vaccinated in all study countries, and we find only three instances where the differences exceed five points (Variable 'Q5' 5.8% in Burkina Faso; 'Q4' 6.2% in Mali; 'Q3' 6.0% in Nigeria; Table A.6). The results from the multivariate logit regression model are also robust to the use of household or individual-level weights. This is true especially of the cross-country findings for urban and richer households and willingness to get tested for COVID-19: the point estimates tend to be slightly larger when using individual-level compared to household-level weights but they are of comparable magnitude and in most cases retain statistical significance (Table A.7). In contrast, the findings on education have similar point estimates whether we use household or individual-level weights, but with individual-level weights they retain statistical significance only in Malawi. This is likely because individual-level weight recalibration increases the variance of the estimates. All in all, we take this set of tests to indicate the robustness of our results to concerns around respondent selection.

Another potential limitation is the possible malleability of attitudes towards vaccinations, which may have changed, and continue to change, in light of the development of various vaccines and the relative success these appear to have in stemming the pandemic in other parts of the world. Similarly, a thorough investigation into the causal factors underlying country- and individual-level differences in COVID-19 acceptance rates is beyond the scope of this paper. Future research is thus needed to determine evolving attitudes in Africa towards being vaccinated, their interactions with vaccine supply and availability, and the driving forces behind variation in acceptance rates at the country and individual level.

Conclusions and policy implications

Our headline results of high vaccine acceptance in a cross-country comparable sample of six Sub-Saharan African countries suggests that inadequate demand is unlikely to represent the key bottleneck to reaching high COVID-19 vaccine coverage in the region. As willingness to be vaccinated does not automatically translate into vaccine-seeking behavior, public authorities need to turn intent into effective demand as vaccine rollout progresses.[41] For this, our study identifies some indicative pockets of hesitancy, particularly in Mali, urban Burkina Faso and Malawi, among women in Nigeria, and for richer households and those with more education. Many of these population groups are comparatively easy to reach early in the vaccine rollout process as well as better reachable through targeted communication. Therefore, information, sensitization, and engagement campaigns that raise acceptance for a COVID-19 vaccine in these clusters and help to maintain the generally high acceptance rates we find will be key. These should focus on

For peer review only

resolving concerns about side effects and bolster confidence in the safety of approved COVID-19 vaccines in order to reach mass coverage and end the pandemic swiftly and everywhere.

Author contributions

All authors had full access to all of the data in the study and take responsibility for the integrity of the data and the accuracy of the data analysis. All authors were responsible for its conception and design. YM, PW, and SK were responsible for the preparation and analysis of the data and all authors contributed to their interpretation. YM and PW drafted the manuscript and AZ made critical revision of the manuscript for important intellectual content. SK and AZ conceptualized an early version. AZ is the guarantor. The corresponding author attests that all listed authors meet authorship criteria and that no others meeting criteria have been omitted.

Funding

Funding for the analysis comes from the World Bank Multi-Donor Trust Fund for Integrated Household and Agricultural Surveys in Low and Middle-Income Countries (TF072496). The funders had no role in study design, data collection, analysis, interpretation of the data, decision to publish, or preparation of the manuscript.

Competing interests

All authors have completed the ICMJE uniform disclosure form at www.icmje.org/coi_disclosure.pdf and declare: no support from any organization for the submitted work; no financial relationships with any organizations that might have an interest in the submitted work in the previous three years; no other relationships or activities that could appear to have influenced the submitted work.

Ethics approval

Each phone survey was implemented by the respective national statistical office (NSO), except for Ethiopia where a private firm was the implementing agency. In Burkina Faso, Malawi, Mali, Nigeria, and Uganda, the NSO conducts the survey as the sole official statistical authority in the country and in accordance with the respective National Statistical Act, which exempts the NSO from institutional ethics approvals. Informed consent was received from all survey respondents in each country. The World Bank does not require institutional ethics approval for household surveys that are partly or fully financed by the World Bank, including the national phone surveys in Burkina Faso, Ethiopia, Malawi, Mali, Nigeria, and Uganda that inform our research.

Data Sharing

The data and country-specific questionnaires are available from the World Bank's Microdata Library, High Frequency Phone Survey Catalog (https://microdata.worldbank.org/index.php/catalog/hfps) as well as LSMS catalog (https://microdata.worldbank.org/index.php/catalog/lsms).

Transparency Statement

The authors affirm that the manuscript is an honest, accurate, and transparent account of the study being reported; that no important aspects of the study have been omitted; and that any discrepancies from the study as planned have been explained.

Dissemination to participants and related patient and public communities:

For peer review only

Our research uses data from a large, anonymized sample of participants and there are thus no plans to disseminate the research to specific participants or patients beyond publishing it.

Licensing

The Corresponding Author is aware that the World Bank external publishing department has recently agreed to grant to BMJ Publishing Group Limited a worldwide, non-exclusive license to publish an article produced by World Bank staff and consultants, and is committed to facilitate the conclusion of a similar agreement should this manuscript be accepted for publication.

Figure captions

Figure 1: Vaccine hesitancy overall, by sex, by residence, and by expenditure quintile

Figure 2: Reasons for covid-19 vaccine hesitancy

For peer review only

References

1 Schellekens P, Indermit G. COVID-19 is a developing country pandemic. Future Development. 2021.https://www.brookings.edu/blog/future-development/2021/05/27/covid-19-is-a-developing-country-pandemic/ (accessed 10 Jun 2021).

2 World Bank. *Poverty and Shared Prosperity 2020 : Reversals of Fortune*. Washington, DC: World Bank 2020. doi:10.1596/978-1-4648-1602-4

3 Josephson A, Kilic T, Michler JD. Socioeconomic impacts of COVID-19 in low-income countries. *Nat Hum Behav* Published Online First: 30 March 2021. doi:10.1038/s41562-021-01096-7

4 Rice BL, Annapragada A, Baker RE, *et al.* Variation in SARS-CoV-2 outbreaks across sub-Saharan Africa. *Nat Med* 2021;**27**:447–53. doi:10.1038/s41591-021-01234-8

5 Britton T, Ball F, Trapman P. A mathematical model reveals the influence of population heterogeneity on herd immunity to SARS-CoV-2. *Science* 2020;**369**:846–9. doi:10.1126/science.abc6810

6 Aschwanden C. Five reasons why COVID herd immunity is probably impossible. *Nature* 2021;**591**:520–2. doi:10.1038/d41586-021-00728-2

7 Schellekens P. For greater vaccine equity, first fix these misconceptions. Future Development. 2021.https://www.brookings.edu/blog/future-development/2021/04/02/for-greater-vaccine-equity-first-fix-these-misconceptions/ (accessed 10 Jun 2021).

8 Berkley S. COVAX explained. #Vaccineswork. 2020.https://www.gavi.org/vaccineswork/covax-explained (accessed 19 May 2021).

9 Wang W, Wu Q, Yang J, *et al.* Global, regional, and national estimates of target population sizes for covid-19 vaccination: descriptive study. *BMJ* 2020;:m4704. doi:10.1136/bmj.m4704

10 Yoo KJ, De Francisco Serpa N, Gordillo-Tobar A. Calculating Sub-Saharan Africa's COVID vaccination financing gap. Investing in Health. 2021.https://blogs.worldbank.org/health/calculating-sub-saharan-africas-covid-vaccination-financing-gap (accessed 19 May 2021).

11 Africa CDC. COVID-19 Vaccine Perceptions: A 15 country study. 2021. https://africacdc.org/download/covid-19-vaccine-perceptions-a-15-country-study/ (accessed 18 May 2021).

12 Seydou A. Who wants COVID-19 vaccination? In 5 West African countries, hesitancy is high, trust low. Afrobarometer 2021. https://bit.ly/3hz1wZg

13 Solís Arce JS, Warren SS, Meriggi NF, *et al.* COVID-19 Vaccine Acceptance and Hesitancy in Low and Middle Income Countries, and Implications for Messaging. Public and Global Health 2021. doi:10.1101/2021.03.11.21253419

14 Bono SA, Faria de Moura Villela E, Siau CS, *et al.* Factors Affecting COVID-19 Vaccine Acceptance: An International Survey among Low- and Middle-Income Countries. *Vaccines* 2021;**9**:515. doi:10.3390/vaccines9050515

15 World Bank. Population, total. World Development Indicators. 2019.https://data.worldbank.org/indicator/SP.POP.TOTL (accessed 15 Jun 2021).

16 Fu H, Sanchez-Paramo C. High-frequency monitoring of COVID-19 impacts. 2020.https://blogs.worldbank.org/opendata/high-frequency-monitoring-covid-19-impacts (accessed 27 Oct 2020).

17 High-Frequency Phone Surveys. World Bank Microdata Library. https://microdata.worldbank.org/index.php/catalog/hfps (accessed 10 May 2021).

18 Ambel A, McGee K, Tsegay A. Reducing Bias in Phone Survey Samples. Effectiveness of Reweighting Techniques Using Face-to-Face Surveys as Frames in Four African Countries. Washington D.C.: : World Bank 2021. https://documents.worldbank.org/en/publication/documents-reports/documentdetail/859261622035611710/reducing-bias-in-phone-survey-samples-effectiveness-of-reweighting-techniques-using-face-to-face-surveys-as-frames-in-four-african-countries

19 Himelein K, Eckman S, Kastelic J, *et al.* High Frequency Mobile Phone Surveys of Households to Assess the Impacts of COVID-19. Guidelines on Sampling Design. Washington D.C.: : World Bank 2020. http://documents.worldbank.org/curated/en/742581588695955271/Guidelines-on-Sampling-Design

20 Himelein K. Weight Calculations for Panel Surveys with Subsampling and Split-off Tracking. *Statistics and Public Policy* 2014;**1**:40–5. doi:10.1080/2330443X.2013.856170

21 Brubaker JM, Kilic T, Wollburg PR. Representativeness of Individual-Level Data in COVID-19 Phone Surveys: Findings from Sub-Saharan Africa. The World Bank 2021. https://econpapers.repec.org/paper/wbkwbrwps/9660.htm (accessed 19 May 2021).

22 Gentilini U, Almenfi M, Blomquist J, *et al.* Social Protection and Jobs Responses to COVID-19: A Real-Time Review of Country Measures (May 14, 2021). Washington D.C.: : World Bank 2021. http://hdl.handle.net/10986/33635

23 Stoop N, Hirvonen K, Maystadt J-F. Institutional mistrust and child vaccination coverage in Africa. *BMJ Global Health* 2021;**6**:e004595. doi:10.1136/bmjgh-2020-004595

For peer review only

24 World Bank, UNDESA. Monitoring the State of Statistical Operations under the COVID-19 Pandemic : Highlights from the Third Round of a Global COVID-19 Survey of National Statistical Offices (NSOs). Washington, D.C.: : The World Bank 2020. http://documents.worldbank.org/curated/en/833751608029512958/Monitoring-the-State-of-Statistical-Operations-under-the-COVID-19-Pandemic-Highlights-from-the-Third-Round-of-a-Global-COVID-19-Survey-of-National-Statistical-Offices-NSOs

25 World Bank, UNDESA. Monitoring the State of Statistical Operations under the COVID-19 Pandemic. Washington, D.C.: : World Bank 2020. https://documents.worldbank.org/en/publication/documents-reports/documentdetail/338481591648922978/monitoring-the-state-of-statistical-operations-under-the-covid-19-pandemic

26 World Bank, UNDESA. Monitoring the State of Statistical Operations under the COVID-19 Pandemic : Highlights from the Second Round of a Global COVID-19 Survey of National Statistical Offices (NSOs). Washington, D.C.: : World Bank 2020. https://documents1.worldbank.org/curated/en/297221597442670485/pdf/Monitoring-the-State-of-Statistical-Operations-under-the-COVID-19-Pandemic-Highlights-from-the-Second-Round-of-a-Global-COVID-19-Survey-of-National-Statistical-Offices.pdf

27 Barello S, Nania T, Dellafiore F, *et al.* 'Vaccine hesitancy' among university students in Italy during the COVID-19 pandemic. *Eur J Epidemiol* 2020;**35**:781–3. doi:10.1007/s10654-020-00670-z

28 Malik AA, McFadden SM, Elharake J, *et al.* Determinants of COVID-19 vaccine acceptance in the US. *EClinicalMedicine* 2020;**26**:100495. doi:10.1016/j.eclinm.2020.100495

29 Al-Mohaithef M, Padhi BK. Determinants of COVID-19 Vaccine Acceptance in Saudi Arabia: A Web-Based National Survey. *JMDH* 2020;**Volume 13**:1657–63. doi:10.2147/JMDH.S276771

30 Skjefte M, Ngirbabul M, Akeju O, *et al.* COVID-19 vaccine acceptance among pregnant women and mothers of young children: results of a survey in 16 countries. *Eur J Epidemiol* 2021;**36**:197–211. doi:10.1007/s10654-021-00728-6

31 Alqudeimat Y, Alenezi D, AlHajri B, *et al.* Acceptance of a COVID-19 Vaccine and its Related Determinants among the General Adult Population in Kuwait. *Med Princ Pract* Published Online First: 22 January 2021. doi:10.1159/000514636

32 Schwarzinger M, Watson V, Arwidson P, *et al.* COVID-19 vaccine hesitancy in a representative working-age population in France: a survey experiment based on vaccine characteristics. *The Lancet Public Health* 2021;**6**:e210–21. doi:10.1016/S2468-2667(21)00012-8

33 Benham B, JH Bloomberg School of Public Health. COVID-19 Vaccine Acceptance Falling Globally and in the U.S., Survey Finds. Johns Hopkins Bloomberg School of Public Health. 2021.https://www.jhsph.edu/news/news-releases/2021/covid-19-vaccine-acceptance-falling-globally-and-in-the-us-survey-finds.html (accessed 26 May 2021).

34 El-Elimat T, AbuAlSamen MM, Almomani BA, *et al.* Acceptance and attitudes toward COVID-19 vaccines: A cross-sectional study from Jordan. *PLoS ONE* 2021;**16**:e0250555. doi:10.1371/journal.pone.0250555

For peer review only

35 Brenan M. Willingness to Get COVID-19 Vaccine Ticks Up to 63% in U.S. Gallup. 2020.https://news.gallup.com/poll/327425/willingness-covid-vaccine-ticks.aspx (accessed 10 Jun 2021).

36 Funk C, Tyson A. Intent to Get a COVID-19 Vaccine Rises to 60% as Confidence in Research and Development Process Increases. Pew Research Center 2020. https://www.pewresearch.org/science/2020/12/03/intent-to-get-a-covid-19-vaccine-rises-to-60-as-confidence-in-research-and-development-process-increases/

37 Neumann-Böhme S, Varghese NE, Sabat I, *et al.* Once we have it, will we use it? A European survey on willingness to be vaccinated against COVID-19. *The European Journal of Health Economics* 2020;**21**:977–82. doi:10.1007/s10198-020-01208-6

38 Lazarus JV, Ratzan SC, Palayew A, *et al.* A global survey of potential acceptance of a COVID-19 vaccine. *Nat Med* 2021;**27**:225–8. doi:10.1038/s41591-020-1124-9

39 Sallam M. COVID-19 Vaccine Hesitancy Worldwide: A Concise Systematic Review of Vaccine Acceptance Rates. *Vaccines* 2021;**9**. doi:10.3390/vaccines9020160

40 Kihara AB. Vaccination against COVID-19 in Africa. *Int J Gynecol Obstet* 2021;**153**:186–7. doi:10.1002/ijgo.13646

41 MacDonald NE. Vaccine hesitancy: Definition, scope and determinants. *Vaccine* 2015;**33**:4161–4. doi:https://doi.org/10.1016/j.vaccine.2015.04.036

For peer review only

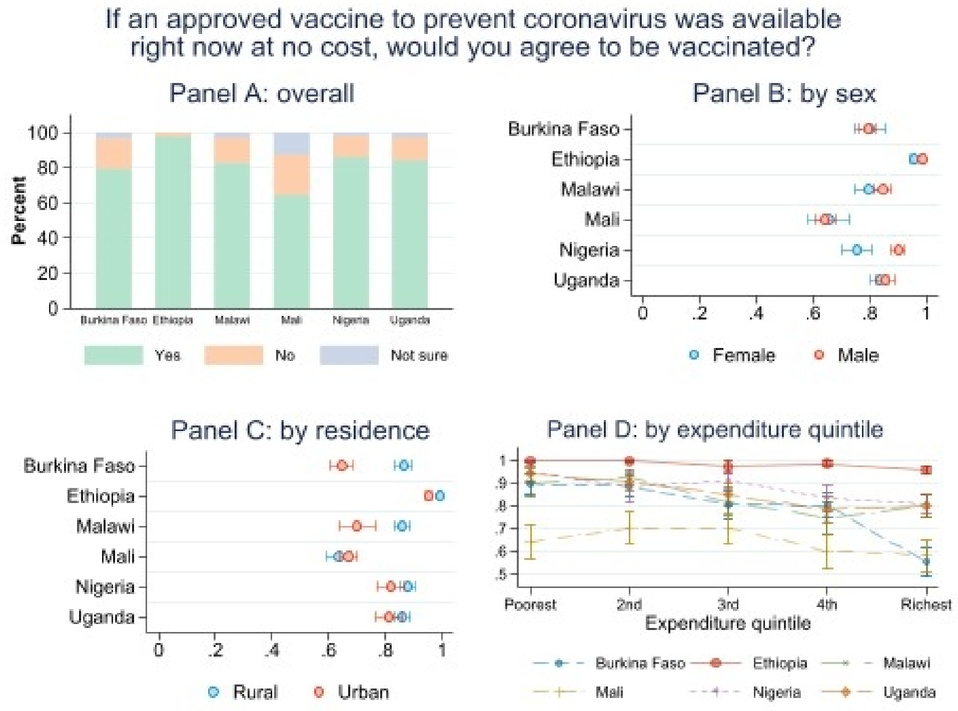

Figure 1: Vaccine hesitancy overall, by sex, by residence, and by expenditure quintile

139x101mm (96 x 96 DPI)

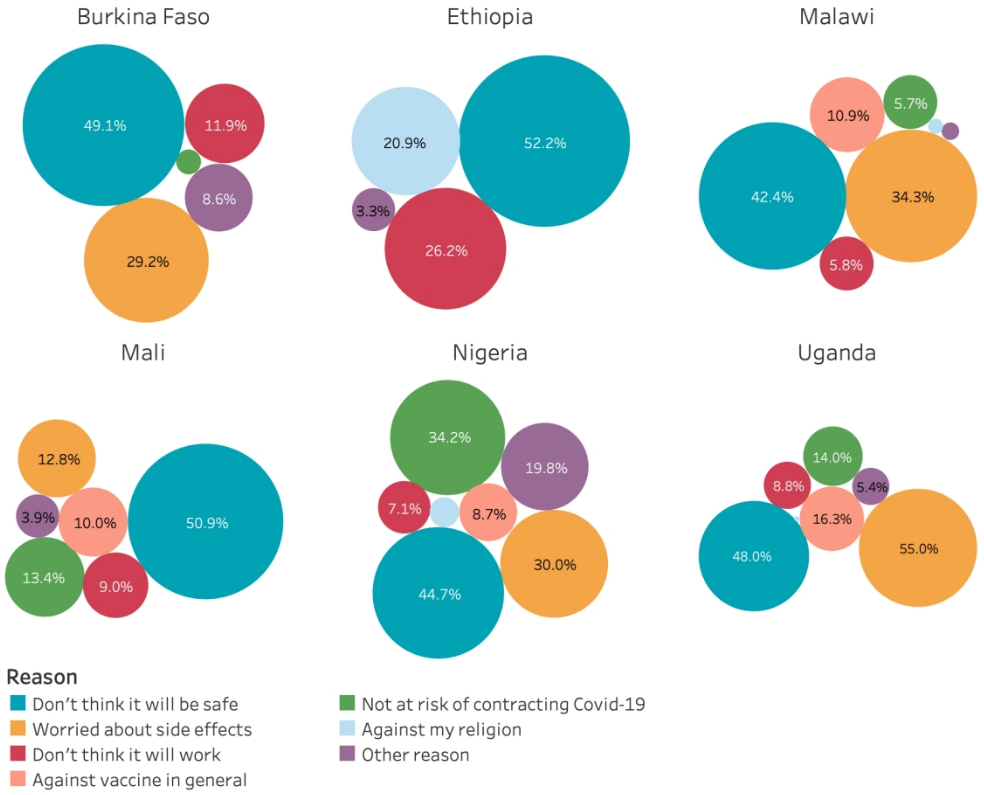

Figure 2: Reasons for covid-19 vaccine hesitancy

321x257mm (144 x 144 DPI)

Appendix

1. Tables

Table A.1. Household selection, number of households

Panel A. Households (N)	**Burkina Faso**	**Ethiopia**	**Malawi**	**Mali**	**Nigeria**	**Uganda**
Pre-COVID-19 FtF survey	7,010	6,770	3,181	7,000	4,976	3,098
With phone numbers	6,877	5,374	2,337	6,300	4,934	2,386
Attempted to contact	2,500	5,374	2,337	2,270	3,000	2,386
Reached	2,062	3,357	1,743	2,270	2,057	2,246
Phone interviews completed	1,945	2,701	1,589	1,765	1,766	2,129

For peer review only

For peer review only - http://bmjopen.bmj.com/site/about/guidelines.xhtml

Table A. 2. Unweighted descriptive statistics in the baseline F2F surveys and the HFPS .

	Burkina Faso		Ethiopia		Malawi		Mali		Nigeria		Uganda	
	F2F	HFPS	F2F	HFPS	F2F	HFPS	F2F	HFPS	F2F	HFPS	F2F	HFPS
Men	45.5	78.7	47.3	62.7	47.6	59.8	47.4	78.9	48.3	72.3	48.2	50.7
HH heads	28.7	86.1	38.5	82.5	37.0	74.5	27.1	82.3	32.7	79.3	35.1	74.5
Urban	44.9	72.0	54.0	71.9	26.0	36.8	41.7	66.3	32.0	39.3	24.2	25.8
Primary education	26.1	32.4	34.6	52.9	37.2	50.2	20.2	30.9	66.5	75.1	47.8	51.1
< 30 years	44.1	11.3	49.6	29.4	52.9	27.8	42.6	13.8	42.5	13.5	48.2	15.3
30 - 60 years	45.7	72.2	42.1	62.4	38.2	62.7	45.7	70.4	44.9	69.0	40.0	66.2
60+ years	10.1	16.5	8.3	8.3	8.9	9.5	11.7	15.9	12.6	17.5	11.8	18.6
Quintile 1 (poorest)	18.7	14.0	11.4	4.6	15.7	8.7	18.2	14.6	12.7	9.5	18.2	15.8
Quintile 2	18.9	15.6	13.8	9.5	17.6	14.6	18.9	16.4	14.4	13.1	19.0	18.5
Quintile 3	19.9	17.9	15.0	12.4	17.4	16.1	21.0	19.0	19.2	18.7	20.4	20.7
Quintile 4	21.3	22.5	20.3	22.0	22.2	23.5	20.7	22.2	21.4	21.9	21.7	22.5
Quintile 5 (richest)	21.2	30.0	39.4	51.5	27.1	37.1	21.2	27.8	32.3	36.9	20.7	22.5
**Observations**	24,396	1,742	17,563	2,701	8,588	1,589	24,394	1,765	15,230	1,766	8,763	2,129

Note: The table shows descriptive statistics and sample sizes for the face-to-face (F2F) pre-COVID samples of the general adult population (>15 years) and the high-frequency phone surveys (HFPS) during the pandemic. All values except observation numbers in percent.

only

For peer review only - http://bmjopen.bmj.com/site/about/guidelines.xhtml

Table A.3. Statistical significance of cross-country differences in acceptance rates in pairwise, bivariate regressions.

**BFA**						
**ETH**	0.000***					
**MWI**	0.089*	0.000***				
**MLI**	0.000***	0.000***	0.000***			
**NIG**	0.000***	0.000***	0.056*	0.000***		
**UGA**	0.005***	0.000***	0.324	0.000***	0.309	
	**BFA**	**ETH**	**MWI**	**MLI**	**NIG**	**UGA**

Note: P-values from bivariate OLS regressions of vaccine acceptance on a single country dummy using samples consisting of each respective country pair. ***p<0.01, **p<0.05, *p<0.1

Table A.4. Willingness to get vaccinated by country and pooled, mean and 95% confidence interval

If an approved vaccine to prevent coronavirus was available right now at no cost, would you agree to be vaccinated? —Yes	Mean	Lower Bound	Upper Bound	N
Burkina Faso, weighted	79.6%	77.0%	82.3%	1,945
Ethiopia, weighted	97.9%	97.2%	98.6%	2,701
Malawi, weighted	82.7%	80.0%	85.4%	1,589
Mali, weighted	64.5%	61.3%	67.8%	1,765
Nigeria, weighted	86.2%	83.9%	88.5%	1,766
Uganda, weighted	84.5%	82.2%	86.8%	2,129
Pooled, weighted	87.6%	86.4%	88.8%	11,895
Pooled, unweighted	82.4%	81.7%	83.1%	

Table A.5: Bivariate logistic regression on government trust and satisfaction using household-level survey weights.

VARIABLES	(1)<br>Malawi	(2)<br>Mali	(3)<br>Nigeria
Government not trustworthy	-0.0638**		
	(0.0322)		
Satisfied with government response		0.161***	0.0568**
		(0.0552)	(0.0232)
Observations	1,589	1,631	1,766
Controls	YES	YES	YES
Pseudo R2	0.00574	0.00742	0.00847

Robust standard errors in parentheses. *** p<0.01, ** p<0.05, * p<0.1

For peer review only - http://bmjopen.bmj.com/site/about/guidelines.xhtml

Table A.6. Estimated acceptance rates using household or individual weights.

	Burkina Faso		Ethiopia		Malawi		Mali		Nigeria		Uganda	
	HH	Indiv.	HH	Indiv.	HH	Indiv.	HH	Indiv.	HH	Indiv.	HH	Indiv.
Overall	0.798	0.815	0.979	0.973	0.827	0.828	0.645	0.657	0.862	0.843	0.845	0.844
	(0.0135)	(0.0176)	(0.0036)	(0.0068)	(0.0137)	(0.0176)	(0.0166)	(0.0241)	(0.0118)	(0.0168)	(0.0116)	(0.0129)
Men	0.800	0.839	0.987	0.987	0.846	0.843	0.642	0.666	0.901	0.888	0.854	0.862
	(0.0151)	(0.0178)	(0.0037)	(0.0050)	(0.0162)	(0.0216)	(0.0185)	(0.0281)	(0.0126)	(0.0200)	(0.0160)	(0.0161)
Women	0.788	0.779	0.958	0.949	0.798	0.816	0.657	0.643	0.757	0.793	0.835	0.831
	(0.0304)	(0.0346)	(0.0088)	(0.0164)	(0.0240)	(0.0268)	(0.0374)	(0.0435)	(0.0269)	(0.0282)	(0.0169)	(0.0191)
Urban	0.653	0.695	0.954	0.947	0.700	0.719	0.669	0.668	0.820	0.814	0.810	0.802
	(0.0207)	(0.0298)	(0.0070)	(0.0131)	(0.0318)	(0.0398)	(0.0156)	(0.0324)	(0.0229)	(0.0302)	(0.0232)	(0.0260)
Rural	0.891	0.905	0.991	0.986	0.859	0.859	0.635	0.650	0.881	0.858	0.861	0.862
	(0.0160)	(0.0181)	(0.0040)	(0.0078)	(0.0148)	(0.0193)	(0.0227)	(0.0335)	(0.0135)	(0.0203)	(0.0132)	(0.0147)
Q1	0.914	0.928	0.997	0.997	0.903	0.922	0.640	0.643	0.949	0.957	0.941	0.956
	(0.0237)	(0.0226)	(0.0023)	(0.0020)	(0.0335)	(0.0280)	(0.0382)	(0.0572)	(0.0244)	(0.0250)	(0.0163)	(0.0120)
Q2	0.914	0.923	0.997	0.994	0.923	0.906	0.703	0.690	0.884	0.849	0.905	0.863
	(0.0224)	(0.0309)	(0.0029)	(0.0055)	(0.0183)	(0.0284)	(0.0347)	(0.0582)	(0.0340)	(0.0493)	(0.0202)	(0.0321)
Q3	0.823	0.812	0.971	0.958	0.819	0.775	0.702	0.699	0.911	0.851	0.849	0.856
	(0.0312)	(0.0406)	(0.0136)	(0.0249)	(0.0329)	(0.0549)	(0.0348)	(0.0552)	(0.0196)	(0.0416)	(0.0263)	(0.0263)
Q4	0.800	0.766	0.986	0.984	0.745	0.779	0.602	0.664	0.834	0.840	0.785	0.784
	(0.0304)	(0.0466)	(0.0048)	(0.0052)	(0.0356)	(0.0400)	(0.0392)	(0.0478)	(0.0286)	(0.0334)	(0.0294)	(0.0324)
Q5	0.548	0.606	0.957	0.949	0.797	0.819	0.582	0.594	0.809	0.798	0.799	0.796
	(0.0312)	(0.0477)	(0.0082)	(0.0171)	(0.0256)	(0.0282)	(0.0361)	(0.0484)	(0.0218)	(0.0304)	(0.0249)	(0.0282)

Note: The table compares estimated acceptance rates for a free, approved COVID-19 vaccine for different demographics. It distinguishes between using weights that correct for coverage and non-response bias at the household-level (household weights) and, where available, using individual-level phone weights that also correct for respondent selection at the individual-level (individual weights). The samples do not comprise observations for which no individual weight could be calibrated due to missing baseline information. Standard errors in parentheses.

For peer review only - http://bmjopen.bmj.com/site/about/guidelines.xhtml

Table A.7. Logistic regression on the correlates of hesitancy using individual-level survey weights, marginal effects.

VARIABLES	(1) Burkina Faso	(2) Ethiopia	(3) Malawi	(4) Mali	(5) Nigeria	(6) Uganda
Respondent is male	0.0265	0.0125	0.0272	0.116**	0.0307	0.00464
	(0.0351)	(0.00942)	(0.0371)	(0.0494)	(0.0406)	(0.0244)
Lives in urban area	-0.0750**	-0.0236**	-0.0930***	-0.0221	-0.00481	0.00853
	(0.0323)	(0.0116)	(0.0348)	(0.0388)	(0.0370)	(0.0223)
Years of education	-0.00462	-0.00154	-0.0123***	-0.00337	-0.00657	-0.00261
	(0.00293)	(0.000938)	(0.00417)	(0.00356)	(0.00410)	(0.00247)
Age group, 30-60 yrs	0.0354	-0.00114	-0.00707	-0.0117	-0.0124	-0.0340
	(0.0490)	(0.00984)	(0.0387)	(0.0476)	(0.0356)	(0.0294)
Age group, 60+ yrs	0.124**	0.00134	-0.0331	-0.0471	-0.0321	0.0134
	(0.0491)	(0.0196)	(0.0720)	(0.0612)	(0.0628)	(0.0317)
Household head	-0.00843	0.0226*	-0.00400	-0.0972*	0.0333	0.0204
	(0.0401)	(0.0119)	(0.0385)	(0.0549)	(0.0415)	(0.0267)
Household Size	0.00287	0.00186	0.00971	-0.00534*	0.00834	0.00268
	(0.00510)	(0.00181)	(0.00842)	(0.00322)	(0.00591)	(0.00564)
Expenditure, 2nd quintile	0.00107	0.00591	-0.0203	0.0270	-0.0994	-0.0871***
	(0.0502)	(0.0131)	(0.0469)	(0.0638)	(0.0757)	(0.0323)
Expenditure, 3rd quintile	-0.0660	-0.0277	-0.178**	0.0325	-0.0371	-0.0813***
	(0.0428)	(0.0193)	(0.0712)	(0.0537)	(0.0666)	(0.0287)
Expenditure, 4th quintile	-0.0921**	-0.00480	-0.118**	-0.0504	-0.0428	-0.122***
	(0.0466)	(0.0118)	(0.0522)	(0.0595)	(0.0588)	(0.0316)
Expenditure, 5th (richest) quintile	-0.123**	-0.0157	-0.0143	-0.115*	-0.0679	-0.103***
	(0.0528)	(0.0126)	(0.0461)	(0.0684)	(0.0631)	(0.0317)
Willing to be tested for COVID-19	0.271***	0.0839***	0.249***	0.468***	0.224***	0.348***
	(0.0287)	(0.0149)	(0.0440)	(0.0171)	(0.0302)	(0.0287)
HH received assistance during COVID-19	0.0840*	0.0177	-0.0412	0.0406	-0.00112	0.0597***
	(0.0505)	(0.0127)	(0.0455)	(0.0598)	(0.0328)	(0.0214)
Gov't not trustworthy			-0.0408			
			(0.0322)			
Satisfied with gov't response				0.0356	0.0235	
				(0.0446)	(0.0325)	
Observations	1,738	2,654	1,542	1,590	1,700	2,106
Pseudo R2	0.225	0.357	0.136	0.325	0.162	0.238

Robust standard errors in parentheses. *** p<0.01, ** p<0.05, * p<0.1. Weighted logit regressions with willingness to take a free, approved COVID-19 vaccine as dependent variable.

For peer review only - http://bmjopen.bmj.com/site/about/guidelines.xhtml

2. Conception and implementation of the High-Frequency Phone Surveys (HFPS)

The High Frequency Phone Surveys (HFPS) from which we take our data were conceived in March 2020 by a working group formed within the World Bank. They are multi-topic, longitudinal surveys consisting of (i) core modules with core questions that are repeated in each survey round, (ii) optional modules for which inclusion was within the discretion of NSOs according to country need and context, (iii) rotating modules with questions that are only periodically featured in specific survey rounds. The questions on vaccine acceptance belong to the latter category of rotating questions and at the time of writing had only been featured once in the fall of 2020.

In order to safeguard cross-country comparability and maintain the same high standard across implementing NSOs, dedicated working groups within the World Bank elaborated standardized guidelines for sampling [1], questionnaire design (guidelines [2], questionnaire template [3], and interviewer manual [4]), and implementation [5]. These guidelines were circulated ahead of the commencement of the surveys and peer-reviewed inside and outside the World Bank.

The countries covered in our study represent all countries that had their HFPS supported by the Living Standards Measurement Study team within the World Bank.[6] These six countries were the ones where rich, pre-COVID, and cross-country comparable data from the LSMS-ISA household surveys that served as sampling frames existed. With regard to the LSMS-supported HFPS, Carletto and Kilic (2020) provide a detailed discussion of their conception and features.[7]

To select respondents for the HFPS, the pre-COVID LSMS-ISA household surveys [8] served as sampling frames. The pre-COVID LSMS-ISA surveys attempted to collect phone numbers from all household members (or alternatively a reference contact such as a neighbor). The sampling for the HFPS started with all enumeration areas covered in the latest pre-COVID LSMS-ISA household survey. Depending on budget availability, either all households with at least one phone number for a household member or reference contact (Ethiopia, Malawi, and Uganda) or a random sub-sample (Burkina Faso, Nigeria, and Mali) was selected for interviewing. Chapter 2.1 of Brubaker et al. (2021) contains a country-by-country discussion of contact protocols.[9] The target sample size for the HFPS was between 1,500 and 1,800 households, a figure that the survey rounds we take our data on vaccine willingness from all meet or exceed (see Table A.1). The target sample size was calculated in order to ensure that, at a minimum, it would be sufficient to detect a 10 percentage point change in the key indicators (COVID-19 knowledge and behavior and labor market impacts) in between rounds with 90% power and 95% confidence at the national level. Notably, sample size requirements for reliable detection of changes across survey rounds are substantially *higher* than what is required for reliable point estimates within a single round as in the case of our study. A more detailed discussion of the sampling process is provided in Himelein et al. (2020).[1]

To ensure smooth implementation and common standards across countries, the launch of the first round of HFPS was proceeded by three days of piloting the questionnaire, CATI technology, survey protocols, and monitoring mechanisms (for more information, see [5]). Questionnaire guidelines and templates were first elaborated by the World Bank's COVID-19 questionnaire working group and served as the backbone of the HFPS implemented in each NSO (see [2–4]). The country-specific questionnaires implemented in the respective survey round from which our

For peer review only - http://bmjopen.bmj.com/site/about/guidelines.xhtml

data on vaccine acceptance stem are openly available together with the data in the World Bank's Micro Data Library.

Interviewers for the survey were selected out of a pool of existing enumerators with experience conducting the LSMS-ISA household surveys. As such, all enumerators had undergone previous LSMS training and were intimately familiar with LSMS-style surveys. In some of the countries, enumerators furthermore had previous experience conducting surveys over the phone. To prepare enumerators specifically for conducting the HFPS, all interviewers received three days of standardized training ahead of the first round of the survey. Additionally, interviewers received a one-day follow-up training in between survey rounds. Regular audio audits ensured consistently high quality between interviewers. The guideline interview manual shared with enumerators across countries can be accessed at [4].

References

1 Himelein K, Eckman S, Kastelic J, *et al*. High Frequency Mobile Phone Surveys of Households to Assess the Impacts of COVID-19. Guidelines on Sampling Design. Washington D.C.: : World Bank 2020. http://documents.worldbank.org/curated/en/742581588695955271/Guidelines-on-Sampling-Design

2 Palacios-Lopez A, Temgoua Noumedem C, Kokas D, *et al*. High Frequency Mobile Phone Surveys of Households to Assess the Impacts of COVID-19 (Vol. 5) : Overview of the Questionnaire Template (English). Washington, D.C.: : World Bank 2020. https://documents.worldbank.org/en/publication/documents-reports/documentdetail/908001588697965879/overview-of-the-questionnaire-template

3 World Bank. High Frequency Mobile Phone Surveys of Households to Assess the Impacts of COVID-19 (Vol. 4) : Questionnaire Template (English). Washington, D.C.: : World Bank 2020. http://documents.worldbank.org/curated/en/567571588697439581/Questionnaire-Template

4 World Bank. COVID-19 CORE-PLUS Questionnaire: Interview Manual. Washington, D.C.: : World Bank 2020. https://documents1.worldbank.org/curated/en/431901588694657348/pdf/Interview-Manual-for-Template-Questionnaire.pdf

5 Amankwah A, Kanyanda S, Illukor J, *et al*. High Frequency Mobile Phone Surveys of Households to Assess the Impacts of COVID-19 (Vol. 3) : Guidelines on CATI Implementation (English). Washington, D.C.: : World Bank 2020. http://documents.worldbank.org/curated/en/189691588696451053/Guidelines-on-CATI-Implementation

6 World Bank. LSMS-Supported High-Frequency Phone Surveys on COVID-19. World Bank. 2020.https://www.worldbank.org/en/programs/lsms/brief/lsms-launches-high-frequency-phone-surveys-on-covid-19 (accessed 4 Aug 2021).

For peer review only - http://bmjopen.bmj.com/site/about/guidelines.xhtml

For peer review only

7 Carletto G, Kilic T. Capitalizing on the World Bank LSMS-ISA Program for High-Frequency Phone Surveys on COVID-19. Washington, D.C.: : World Bank 2020. https://thedocs.worldbank.org/en/doc/772531587652004382-0050022020/original/ProposalCapitalizingontheLSMSISAProgramforHighFrequencyPhoneSurveysonCOVID19.pdf

8 World Bank. About LSMS. LSMS. https://www.worldbank.org/en/programs/lsms/overview (accessed 4 Aug 2021).

9 Brubaker JM, Kilic T, Wollburg PR. Representativeness of Individual-Level Data in COVID-19 Phone Surveys: Findings from Sub-Saharan Africa. The World Bank 2021. https://econpapers.repec.org/paper/wbkwbrwps/9660.htm (accessed 19 May 2021).

For peer review only

For peer review only - http://bmjopen.bmj.com/site/about/guidelines.xhtml

Reporting checklist for cross sectional study.

Based on the STROBE cross sectional guidelines.

von Elm E, Altman DG, Egger M, Pocock SJ, Gotzsche PC, Vandenbroucke JP. The Strengthening the Reporting of Observational Studies in Epidemiology (STROBE) Statement: guidelines for reporting observational studies.

For peer review only

		Reporting Item	Page Number
**Title and abstract**			
Title	#1a	Indicate the study's design with a commonly used term in the title or the abstract	1
Abstract	#1b	Provide in the abstract an informative and balanced summary of what was done and what was found	1,2
**Introduction**			
Background / rationale	#2	Explain the scientific background and rationale for the investigation being reported	3
Objectives	#3	State specific objectives, including any prespecified hypotheses	1,3
**Methods**			
Study design	#4	Present key elements of study design early in the paper	3-5

Setting	#5	Describe the setting, locations, and relevant dates, including periods of recruitment, exposure, follow-up, and data collection	4
Eligibility criteria	#6a	Give the eligibility criteria, and the sources and methods of selection of participants.	4-5
	#7	Clearly define all outcomes, exposures, predictors, potential confounders, and effect modifiers. Give diagnostic criteria, if applicable	4
Data sources / measurement	#8	For each variable of interest give sources of data and details of methods of assessment (measurement). Describe comparability of assessment methods if there is more than one group. Give information separately for for exposed and unexposed groups if applicable.	4-5
Bias	#9	Describe any efforts to address potential sources of bias	5
Study size	#10	Explain how the study size was arrived at	4-5
Quantitative variables	#11	Explain how quantitative variables were handled in the analyses. If applicable, describe which groupings were chosen, and why	5
Statistical methods	#12a	Describe all statistical methods, including those used to control for confounding	5
Statistical methods	#12b	Describe any methods used to examine subgroups and interactions	5

For peer review only

Statistical methods	#12c	Explain how missing data were addressed	18
Statistical methods	#12d	If applicable, describe analytical methods taking account of sampling strategy	5
Statistical methods	#12e	Describe any sensitivity analyses	5
**Results**			
Participants	#13a	Report numbers of individuals at each stage of study—eg numbers potentially eligible, examined for eligibility, confirmed eligible, included in the study, completing follow-up, and analysed. Give information separately for for exposed and unexposed groups if applicable.	16
Participants	#13b	Give reasons for non-participation at each stage	16
Participants	#13c	Consider use of a flow diagram	n/a
Descriptive data	#14a	Give characteristics of study participants (eg demographic, clinical, social) and information on exposures and potential confounders. Give information separately for exposed and unexposed groups if applicable.	17
Descriptive data	#14b	Indicate number of participants with missing data for each variable of interest	n/a

For peer review only

Outcome data	#15	Report numbers of outcome events or summary measures. Give information separately for exposed and unexposed groups if applicable.	16
Main results	#16a	Give unadjusted estimates and, if applicable, confounder-adjusted estimates and their precision (eg, 95% confidence interval). Make clear which confounders were adjusted for and why they were included	6,16
Main results	#16b	Report category boundaries when continuous variables were categorized	8
Main results	#16c	If relevant, consider translating estimates of relative risk into absolute risk for a meaningful time period	n/a
Other analyses	#17	Report other analyses done—e.g., analyses of subgroups and interactions, and sensitivity analyses	7-8,10-11, 18-20
**Discussion**			
Key results	#18	Summarise key results with reference to study objectives	9
Limitations	#19	Discuss limitations of the study, taking into account sources of potential bias or imprecision. Discuss both direction and magnitude of any potential bias.	10-11
Interpretation	#20	Give a cautious overall interpretation considering objectives, limitations, multiplicity of analyses, results from similar studies, and other relevant evidence.	11

For peer review only

Generalisability	#21	Discuss the generalisability (external validity) of the study results	10-11
**Other Information**			
Funding	#22	Give the source of funding and the role of the funders for the present study and, if applicable, for the original study on which the present article is based	12

Notes:

- 17: 7-8,10-11, 18-20 The STROBE checklist is distributed under the terms of the Creative Commons Attribution License CC-BY. This checklist was completed on 22. June 2021 using https://www.goodreports.org/, a tool made by the EQUATOR Network in collaboration with Penelope.ai

For peer review only
